# Artificial Intelligence-Assisted Systematic Literature Reviews in Metabolic Phenotyping: A Methodological Framework and Validation Study

**DOI:** 10.1007/s43657-026-00336-y

**Published:** 2026-08-17

**Authors:** Maartje Cox, Javier Osorio Mosquera, Shaimaa Khandaker, Samuele Sala, Julien Wist, Jeremy K. Nicholson, Elaine Holmes, Timothy Fairchild, Nathan G. Lawler

**Affiliations:** 1 Australian National Phenome Centre, Health Futures Institute, Harry Perkins Institute of Medical Research, Murdoch University, 5 Robin Warren Drive, Murdoch, Perth, WA 6150 Australia; 2 Chemistry Department, Universidad del Valle, Melendez, 76001 Cali, Colombia; 3 Centre for Computational and Systems Medicine, Health Futures Institute, Harry Perkins Institute, Murdoch University, Perth, WA 6150 Australia; 4 Department of Metabolism, Digestion and Reproduction, Faculty of Medicine, Imperial College, London, SW7 2AZ UK; 5 School of Allied Health, College of Health and Education, Murdoch University, Perth, WA 6150 Australia; 6 Centre for Healthy Ageing, Health Futures Institute, Murdoch University, Perth, WA 6150 Australia

**Keywords:** Systematic Literature Reviews, Artificial Intelligence, Metabolomics, Long COVID

## Abstract

Systematic literature reviews are widely acknowledged to be at the top of most evidence hierarchies but are very time-consuming and labour-intensive. The advent of artificial intelligence (AI) offers possibilities to accelerate the review process. We propose a systematic literature review protocol that incorporates AI and evaluate the accuracy of AI against a human-constructed gold standard. Human researchers performed a systematic literature review on the PubMed metabolomic literature investigating Long COVID. OpenAI model o3-mini was then used via the application programming interface to carry out the three key stages of a systematic literature review: 1) screening articles for inclusion criteria, 2) evaluating articles' risk of bias (RoB) using a customised metabolomics tool based on well-established quality criteria, and 3) extracting data from articles. The performance of AI was evaluated against a manually created reference by the assessment of accuracy, negative predictive value, precision, sensitivity, specificity, F1 score, and Cohen's kappa. Out of the 320 identified articles, 33 met all inclusion criteria. AI performed screening, RoB assessment, and data extraction with mean accuracies of 92.7% ± 4.9%, 91.9% ± 5.9%, and 92.0% ± 9.2%, respectively, in ~ 10 h and at a cost of ~ 15.35 USD, while human reviewers took ~ 116 h, equivalent to ~ 2000 USD at minimum wages, to complete these steps in duplicate. OpenAI model o3-mini can independently complete the three main stages of systematic literature reviews in the metabolomics domain, thus considerably reducing the time to complete such reviews, at minimal cost. Nonetheless, human reviewers should always verify the AI-generated output to ensure robustness.

**Supplementary Information:**

The online version contains supplementary material available at https://doi.org/10.1007/s43657-026-00336-y.

## Introduction

Systematic literature reviews have been recognised as one of the highest levels of scientific evidence in the medical literature (Wallace et al. 2022), as they provide a rigorous and comprehensive overview of all evidence collected on a particular topic. Furthermore, they are of interest to a wide audience, including medical doctors, researchers, patients, and policy makers (Sriganesh et al. 2016; López-Pineda et al. 2025). To classify as "systematic", literature reviews must be conducted according to well-established guidelines such as the Preferred Reporting Items for Systematic Reviews and Meta-Analyses (PRISMA) statement to ensure transparency, accuracy, completeness and usefulness for users (Page et al. 2021).

Systematic literature reviews can be extremely insightful in the field of metabonomics (also known as metabolomics or metabolic phenotyping), which analyses metabolites that represent the substrates, intermediates, and products of cellular metabolism (Johnson et al. 2016), and often compares metabolite levels between patients and healthy individuals. The types and amounts of metabolites detected in samples depend heavily on the analytical platform(s) used, which have different strengths, limitations, and biases and can vary between laboratories. Consequently, the metabolomics literature encompasses a vast range of metabolites found to be associated with specific acute or chronic diseases, such as acute coronavirus disease (COVID)-19, sepsis and septic shock, rheumatoid arthritis, and type 2 diabetes (Huffman et al. 2017; Yang et al. 2018; Lodge et al. 2023, 2024; Cox et al. 2025). Systematic literature reviews of metabolic phenotyping studies can comprehensively summarise all metabolites significantly affected by disease and allow for research findings to be validated between various research facilities. For instance, one study systematically reviewed prospective metabolic studies related to dementia and formed a comprehensive list of metabolites—including amino acids, lipids, and lipoproteins—with prognostic potential for dementia onset (Flores et al. 2024), another study explored metabolic characteristics of patients with varying degrees of acute COVID-19 infection severity based on the results of 21 studies (Bi et al. 2025), and yet another study identified five metabolites as potential rheumatoid arthritis biomarkers based on their coherence across studies after meta-analysing 49 articles (Song et al. 2024). Another advantage of systematically reviewing the metabolomics literature is the opportunity it provides to critically evaluate the reporting of analytical practices against well-established reporting standards, e.g., those from the Metabolomics Standards Initiative (MSI) (Sumner et al. 2007) and the Metabolomics Quality Assurance and Quality Control Consortium (MQACC) (Kirwan et al. 2022), during risk of bias (RoB) assessment.

Although the benefits of systematic literature reviews are clear, a well-recognised disadvantage is that the process is resource- and time-consuming, with an average of five authors involved and a time window from project registration to publication of up to 186 weeks (67.3 ± 31.0 weeks across 192 publications) (Borah et al. 2017). In particular, steps such as screening large bodies of literature for pre-defined inclusion criteria, extracting data from included articles, assessing their RoB, and synthesising the results take a considerable amount of time. This is doubled when these processes are performed independently by two or more reviewers and even more time is invested when interference of an independent person is required to resolve discrepancies between the decisions from the first and second reviewers. 

The introduction of large language models (LLMs) such as ChatGPT, DeepSeek, Claude, and Gemini has opened avenues to speed up the systematic literature review process and reduce the number of human reviewers involved (Alshami et al. 2023; López-Pineda et al. 2025; Clark et al. 2025). A growing body of literature has explored the use of Artificial Intelligence (AI) in various stages of the systematic literature review process, which included searching literature of interest, screening articles for inclusion criteria (Syriani et al. 2024; López-Pineda et al. 2025), performing RoB assessment, and extracting data. Studies have accomplished these tasks with varying degrees of success (Blaizot et al. 2022). For instance, a recent systematic literature review by Clark et al. (2025) demonstrated that using AI to search relevant literature resulted in 68%-96% of studies being missed. The percentages for incorrect inclusion and exclusion decisions, incorrect data extraction, and incorrect RoB assessment were lower (i.e., medians of 10%, 28%, 14%, and 27%, respectively), yet still indicate a considerable error rate that may impact the accuracy of systematic literature reviews (Clark et al. 2025). Similarly, Eisele-Metzger et al. (2025) found an agreement of 41% between the LLM Claude 2 and human reviewers in assessing the RoB of 100 randomised controlled trials (RCTs). On the contrary, AI models Llama 3 and GPT-4o mini yielded an accuracy rate > 90% in screening titles and abstracts (López-Pineda et al. 2025). The large variability in the accuracy of AI's responses is arguably the largest, albeit not the only, limitation. A second limitation is that AI’s performance strongly depends on the quality of the input data (i.e., the prompt with relevant text extracted from a paper), with unclear, unstructured, and/or unspecific prompts and incomplete information resulting in responses of poorer quality (Alshami et al. 2023; Mahuli et al. 2023; Giray 2023; Mostafapour et al. 2024). Thirdly, there are several ethical concerns associated with the use of AI, such as the accountability for AI-generated results and the appropriate contextualisation thereof in the larger framework of scientific knowledge (Alshami et al. 2023; Haman and Školník 2023). Because of these limitations, several studies concluded that AI is currently not sufficiently accurate to independently perform systematic literature reviews, but is well able to assist and improve efficiency under the oversight of human researchers (Blaizot et al. 2022; Alshami et al. 2023; Mahuli et al. 2023; Mostafapour et al. 2024; López-Pineda et al. 2025; Clark et al. 2025). In line with these recommendations, we here propose a systematic literature review protocol that integrates the power of AI into the traditional workflow and includes a new RoB assessment tool tailored to the critical evaluation of metabolomics methods. The novelty of this protocol lies in the usage of AI for all three key stages of the literature review process (i.e., 1. screening articles for inclusion criteria, 2. evaluating articles’ RoB using a customised metabolomics tool based on well-established quality criteria, and 3. extracting data from articles), and the usage of AI to evaluate the RoB of metabolomics papers in particular. We demonstrate this innovative workflow using the PubMed literature that investigated the metabolome of Long COVID patients and determined the accuracy of AI in screening articles for pre-defined inclusion criteria, assessing RoB, and extracting data from articles. While this study illustrates the novel workflow with several domain-specific elements (including a metabolomics-adapted RoB tool and Long COVID use case), the general idea of a hybrid human-AI workflow for systematic literature reviews is domain-agnostic and therefore adaptable to other fields.

## Methods

### Approach

A systematic review of the metabolomics literature on Long COVID was conducted by human researchers, without the involvement of AI. Subsequently, AI was queried to perform three key stages of the review process: 1) screening articles for eligibility, 2) performing RoB assessment, and 3) extracting data from included articles. The following paragraphs briefly describe the traditional/manual and automated review processes while specifications of all steps are provided in the next sections.

#### Traditional Literature Review

This review was registered in the Open Science Framework (OSF; https://doi.org/10.17605/OSF.IO/H9P84). A human researcher (MC) searched the PubMed literature, performed manual forward citation to retrieve further articles of interest, screened articles for pre-defined inclusion criteria, performed RoB assessment, and extracted key details from the included studies. The results from screening, RoB assessment, and data extraction were checked and, if necessary, corrected by a second reviewer (SK or SS) to create manually curated gold standards against which the AI responses were tested. Conflicts between responses of the first and second reviewers were resolved through discussion and with the help of a third reviewer (NGL) if disagreements could not be resolved.

#### Artificial Intelligence-Based Literature Review

Prompts were developed, tested, and optimised to query AI for performing the three key stages of the review process. In line with previously published recommendations (Giray 2023), prompts are composed of clearly formulated criteria or questions (which should be evaluated by the model and for which responses should be provided), specific instructions detailing the task that AI is requested to perform, and an output indicator providing the structure in which AI should format the output. Additionally, the prompt either includes the articles' abstracts (Prompt 1 and 2, Supplementary Information (SI) S1-2) or provides the full-text PDF as an attachment (Prompt 3–14, SI S3-14), providing sufficient knowledge for the model to generate an accurate response. Supplementary files were provided to AI as attachments if crucial information was not provided in the main article, e.g., if a detailed description of the (analytical) methods was exclusively provided in a supplementary file. Prompts were engineered by MC and optimised using OpenAI model GPT-4.1 mini. Pilot tests were conducted with OpenAI models GPT-4o, o3-mini, and o1-preview. Manual inspection of the test results led to further optimisation of the prompts and to the selection of the o3-mini model for the purpose of this study. Given the rapid pace of AI development, this study did not focus on benchmarking multiple AI models. Instead, the framework presented here was designed to be model-agnostic and applicable to different AI models as they evolve. All prompts are provided in the supplementary file (SI S1-14).

Results from AI were compared to the gold standard created by human reviewers to determine the accuracy of AI. The OpenAI model o3-mini was queried through the application programming interface (API). For prompts without attachments (i.e., abstracts), the OpenAI's API Create Completion was used, with the following request body parameters (inference configuration parameters): 1) model; 2) messages (i.e., the prompt); 3) temperature = 1 (API default); 4) maximum number of tokens generated in the completion (max_tokens = 9152); 5) top_p = 1 (API default); 6) frequency_penalty = 0 (API default); 7) presence_penalty = 0 (API default) (OpenAI 2025a). For prompts with full-text PDFs as attachments, the query process involved uploading the file to a vector store, creating a temporary assistant with file search enabled, initiating a thread with the prompt linked to the PDF, and waiting for completion. A 30-s delay was introduced manually ——beyond the API parameters ——to prevent failed queries when running the threads with PDF attachments (OpenAI 2025b). Estimates of the costs associated with running the prompts were obtained from OpenAI's API Platform, which also reported the number of input and output tokens, the latter offering a more objective measure of costs since charges are determined by token usage rather than query count.

### Literature Search

Evidence from previous research has cautioned against the use of Generative AI to search for articles by itself (Clark et al. 2025). Therefore, the literature search was performed by a human researcher (MC). To retrieve scientific articles employing a metabolomics approach to investigate Long COVID, the PubMed database was searched on 13 March 2025 with a filter to only search for articles published between 1 October 2019 and 13 March 2025. The search string is provided in the supplementary file (SI S15). Manual forward citation was performed to recover further articles of interest.

### Screening Studies for Eligibility

Articles were deemed eligible for inclusion in this review if they met the following inclusion criteria: 1) the article was written in English; 2) the study was an original investigation that presented new data; 3) the study investigated human participants; 4) the population investigated in the study consisted of at least Long COVID patients and healthy controls, or Long COVID patients and individuals who fully recovered from infection with Severe Acute Respiratory Syndrome Coronavirus 2 (SARS-CoV-2); and 5) the study analysed the metabolic phenotype of the study population. Inclusion criteria one, two, and three were assessed based on the articles' abstracts, while criteria four and five were evaluated based on the full-text articles. Articles were excluded if they 1) were literature reviews; 2) only used publicly available or previously published data; or 3) investigated animals, plants, cell cultures, or other non-human subjects. Articles meeting all inclusion criteria were manually evaluated to determine whether statistically meaningful comparisons of metabolite levels were made between Long COVID patients and healthy controls or recovered individuals. If this was not the case, or if the level of statistical significance was not reported, the article was excluded despite meeting all inclusion criteria.

AI screened articles for these inclusion criteria in three stages. In the first stage, titles and abstracts of articles were parsed to OpenAI, accompanied by Prompt 1 (Original Research Prompt, SI S1), which requests the model to check whether the study was an original investigation. Titles and abstracts of studies not meeting this criterion were removed from the database, and titles and abstracts of the remaining studies were parsed again in the second stage, together with Prompt 2 (Human Participants Prompt, SI S2), which queries the model to evaluate whether the study investigated humans. Studies meeting this criterion proceeded to the third stage, in which the manually retrieved full-text PDFs of these articles were parsed together with Prompt 3 for the evaluation of the remaining criteria (Inclusion Criteria Prompt, SI S3).

### Risk of Bias Assessment

An RoB assessment tool was adapted from U et al. (2022), with several modifications to evaluate the RoB of the studies under review. The RoB assessment tool consists of four domains: 1) study design, 2) chemical analysis, 3) data analysis, and 4) metabolite identification (Table 1). For each item, pre-defined categorical answers were provided, which were labelled as low, medium, or high RoB, or as N/A if the item did not apply to the study under evaluation. A detailed overview of the modifications made to the tool from U et al. (2022) is provided in the Supplementary File (SI S16). The first domain was adjusted for applicability to Long COVID populations, with criteria evaluating the number of Long COVID cases, the reporting of Long COVID symptom duration, the confirmation of SARS-CoV-2 infection through polymerase chain reaction (PCR), rapid antigen, or antibody testing, and the absence of ongoing COVID-induced symptoms in individuals defined as "recovered" from their SARS-CoV-2 infection (Table 1, Domain 1). In the second domain, the reporting of chemical analysis was assessed, including the method of sample collection (item 2.2) and the sample processing method (item 2.3). For these items, "Yes" was answered if at least one collection-related or processing-related item is reported in the article, respectively. Furthermore, the second domain considered the reporting of the sample type used for metabolomics analysis (item 2.1), the separation method (item 2.6), and the instrument type for mass spectrometry and nuclear magnetic resonance (NMR) spectroscopy studies (items 2.7 and 2.8). The second domain is not applicable to studies employing combined non-invasive Magnetic Resonance Spectroscopy/Imaging (MRS/MRI) methods, as no biological samples are collected in these studies. Items in the third domain "Data Analysis" have not been altered from the original tool by U et al. (2022), while for the fourth domain, "Metabolite Identification", only the most important item was retained, i.e., the evaluation of the confidence in metabolite identification according to the well-established guidelines from the MSI (Sumner et al. 2007). The RoB levels "High", "Medium", and "Low" were assigned scores of -1, 0, and 1, respectively.

**Table 1:** Risk of bias assessment tool for applied metabolomics studies

Item		Category	Risk of bias	Prompt (criterion)
Domain 1 – Study Design				
1.1	Number of Long COVID cases	*n* ≥ 50 10 ≤ *n* < 50 *n* < 10	Low Medium High	Cohort RoB (longCovidNumber)
1.2	Did the study validate their metabolomics results using an independent internal or external validation cohort (i.e., was there a discovery and validation cohort)?	Yes No	Low Medium	Reporting RoB (validationCohort)
1.2a	Are participant characteristics (e.g., age, BMI, sex) reported by study group (e.g., Long COVID, recovered individuals, healthy controls)?	Yes No	Low High	Reporting RoB (characteristicsReported)
1.2b	If the study performed validation using an independent (internal or external) cohort, are participant characteristics (e.g., age, BMI, sex) reported for each cohort (i.e., discovery and validation cohort)?	Yes No Not applicable	Low High NA	Reporting RoB (characteristicsPerCohort)
1.3	Long COVID symptom duration	≥ 2 months < 2 months or not reported	Low High	Cohort RoB (symptomDuration)
1.4	Was SARS-CoV-2 infection in Long COVID patients confirmed with a positive PCR, rapid antigen, or antibody test?	Yes No or not reported	Low High	Cohort RoB (infection)
1.5	If the study included a group of individuals who recovered from SARS-CoV-2 infection, was their infection confirmed by a positive PCR, rapid antigen, or antibody test?	Yes No Not applicable	High Low NA	Cohort RoB (definitionRecovered)
1.6	If the study included a group of individuals who recovered from SARS-CoV-2 infection, is it specified that these participants did not experience any remaining symptoms from their infection at the time of study participation?	Yes No Not applicable	Low Medium NA	Cohort RoB (recovered)
1.7	Are inclusion and exclusion criteria stated per study group?	Stated per group Not stated per group	Low Medium	Reporting RoB (inclusionCriteria)
1.8	Were potential confounders (e.g., sex, age) discussed and taken into account in data analysis?	Discussed and taken into account by adding confounders to models or by stratifying analyses Discussed Not discussed	Low High High	Reporting RoB (confoundersReported)
Domain 2 – Chemical Analysis*				
2.1	Is the sample type reported?	Yes No Not applicable	Low High NA	Analysis RoB (sampleType)
2.2	Is the method of biofluid or tissue collection described?	Yes, by describing at least one of the following: collection method (e.g., venipuncture, finger prick, biopsy needle), collection tubes (e.g., EDTA or heparin), and/or anticoagulant use No Not applicable	Low High NA	Analysis RoB (sampleCollection)
2.3	Is the sample processing method described?	Yes, by describing at least one of the following: centrifugation, use of solvents or buffers, resolubilisation, derivatisation, resuspension, use of internal standards or calibrants, and/or tissue handling methods such as pulverisation/homogenisation and lyophilisation No Not applicable	Low High NA	Analysis RoB (samplePreparation)
2.4	Were samples randomised prior to analyses?	Yes Not stated Not applicable	Low Medium NA	Samples RoB (randomisation)
2.5	Was a pooled quality control sample used and its reproducibility shown?	Used and reproducibility shown Used – reproducibility not shown Not reported Not applicable	Low Medium High NA	Samples RoB (qualityControl)
2.6	Was the separation method reported?	Yes, the chromatography type is reported No Not applicable	Low High NA	Analysis RoB (separation)
2.7	If the study used mass spectrometry, was the instrument type reported?	Yes, the type of mass spectrometer is reported (e.g., triple quadrupole, time of flight, ion trap, orbitrap) No Not applicable	Low High NA	Analysis RoB (massSpec)
2.8	If the study used (nuclear) magnetic resonance spectroscopy or magnetic resonance imaging, was the instrument's magnetic field strength and/or proton resonance frequency described?	Yes, the magnetic field strength and/or proton resonance frequency is described No Not applicable	Low High NA	Magnet RoB (magnet)
**Domain 3 – Data Analysis**				
3.1	Is the data analysis workflow clearly described?	Clearly described how data were processed and which (statistical) methods were used to analyse the data. Detailed enough to reproduce the workflow Some information regarding the data analysis workflow is provided, but there are uncertainties and the level of detail is insufficient to reproduce the data analysis workflow No information regarding the data analysis workflow is provided at all	Low Medium High	Samples RoB (dataAnalysis)
3.2	Was an established software/algorithm used to analyse data and if not, is the software or algorithm provided and validated for the new analysis?	Yes, established software was used or newly developed software or algorithm is provided and validated for the new data analysis No, new software or algorithm was used but information provided on software or algorithm was incomplete and/or not validated No, new software or algorithm was used but no information on software or algorithm was provided at all and/or the new software or algorithm was not validated	Low Medium High	Samples RoB (software)
3.3	Were the levels of significance corrected for multiple testing (e.g., with Bonferroni or Benjamini–Hochberg correction)?	Yes No	Low High	Samples RoB (significanceLevel)
**Domain 4 – Metabolite Identification**				
4.1	Level of confidence in metabolite identification **	1 – identified 2 – putatively annotated 3 – putatively characterised compound class 4 – unknown	Low Medium High High	Metabolites RoB (metaboliteIdentificationLevel)

^*^ Items in Domain 2 are not applicable to studies employing combined non-invasive Magnetic Resonance Spectroscopy (MRS) and Magnetic Resonance Imaging (MRI) methods

^**^ Minimum metadata relative to metabolite identification (Sumner, et al. 2007)

Previous results indicated that AI, specifically LLMs, were not able to replace human RoB assessments, but recommended further exploration of the potential of LLMs to support RoB assessment (Eisele-Metzger et al. 2025). In this method, we have done so by creating customised prompts based on the newly developed RoB assessment tool, which allow for an evaluation of all RoB items by AI per article included in the review. Altogether, six prompts were created to cover all 22 RoB items (Cohort RoB, Reporting RoB, Metabolites RoB, Samples RoB, Analysis RoB, Magnet RoB, SI S4-9). Table 1 indicates the prompt that evaluates each specific item.

### Data Extraction

The following descriptive parameters were extracted from the full-text of all included articles: 1) study design (e.g., cross-sectional or longitudinal case–control); 2) country in which the study was conducted; 3) group names; 4) number of participants per group; 5) symptoms reported by Long COVID patients; 6) number of Long COVID patients reporting each symptom; 7) percentage of Long COVID patients reporting each symptom; 8) symptom severity metric; 9) symptom severity (central tendency or qualitative description); 10) symptom severity (standard deviation, interquartile range, or range); 11) significance of the comparison of symptom severity between patients and comparison group; 12) identification of study groups and demographics per group; 13) age, 14) sex, 15) height, 16) weight, 17) BMI; 18) identification of Long COVID patient group and/or recovered group and infection history for each of these groups; 19) the amount of time elapsed between infection and participation in the study as well as 20) the number of times they were infected; 21) the sample type collected from Long COVID patients for metabolomics analyses (e.g., plasma, serum, urine, muscle tissue); 22) whether the study adopted a targeted or untargeted metabolomics approach; and 23) the analytic platform(s) that was/were used for metabolomics analyses. For any parameters not reported in articles or their supplementary materials, NA was noted.

In order to query AI to extract these parameters from the full-text PDFs of the included articles, the 23 parameters were divided into five domains. Domain one contained items 1–4; domain two items 5–11; domain three items 12–17; domain four items 18–20; and domain five items 21–23. Five separate prompts were created accordingly (Design Prompt, Symptoms Prompt, Demographics Prompt, Infection History Prompt, Sample Analysis Prompt, SI S10-14). If parameters could not be extracted from the main text because they were exclusively reported in appendices, then these supplementary materials were parsed to AI as well.

### Determination of the Accuracy of Artificial Intelligence in Screening Articles, Performing Risk of Bias Assessment, and Extracting Data from Articles

For the quantitative and categorical responses (i.e., numerical 1/-1/0 responses and categorical Yes/No) from the screening and RoB stages, the human-extracted reference data were automatically compared to AI answers. For the qualitative responses from the data extraction stage, AI answers were manually contrasted to the gold standard answers. The percentage of accuracy was determined per item, criterion, or parameter. For each inclusion criterion, precision, negative predictive value, sensitivity, specificity, F1, and Cohen's kappa statistics were also determined to provide further insights into the agreement between AI and the gold standard answers. Precision provides insight into how many of AI's "Yes" responses were deemed "Yes" according to the gold standard, while the negative predictive value provides similar insights for “No” responses. Sensitivity and specificity identify how many of the gold standard's "Yes" and "No" answers, respectively, were correctly labelled as such by AI, while the F1 statistic is the harmonic mean of precision and sensitivity. Finally, Cohen's kappa (κ) was calculated as a metric for inter-rater reliability for all inclusion and RoB criteria, and κ valuestatistics were interpreted according to previously published guidelines (Landis and Koch 1977). It must be noted that AI responses of "Not sure" and "Not reported" were not considered for the calculation of any metrics except κ.

### Multidimensional Evaluation of Artificial Intelligence's Mistakes

All mistakes made by AI in comparison to the manually created reference were systematically categorised into one or more of the following seven categories using a multidimensional evaluation approach: 1. comprehension error (i.e., AI misunderstood or misinterpreted information in the abstract or article), 2. information omission error (AI missed relevant data or evidence that was present in the source), 3. hallucination error (AI invented data, claiming that it was reported in the source while in fact it was not ——information provided by AI was not factually correct), 4. inference error (**a**. incorrect inference drawn from the text beyond what was explicitly stated, **b**. failure to make correct implicit inference), 5. instruction-following error (AI failed to correctly apply the task instructions as indicated in the prompt), 6. irrelevance error (AI's response did not address all aspects of the prompt and the user intent), and 7. source-related error (AI did not have access to the information required to provide a correct answer).

## Results

### Literature Search

The PubMed search recovered 321 articles, and forward citation yielded one paper of interest, bringing the total to 322 articles to be screened for eligibility. AI yielded an overall accuracy of 92.0% ± 7.5% across the screening, RoB, and data extraction stages. Further accuracy and inter-rater reliability metrics are provided per stage in the following sections. Manually extracted results and raw results obtained from AI per prompt are made available in the OSF repository (https://doi.org/10.17605/OSF.IO/H9P84), as well as an overview of the incorrect responses generated by AI per prompt.

### Screening Studies for Eligibility

Two articles were excluded because they were not written in English. Abstracts of the remaining 320 articles were screened for the original research and human study population criteria. Of these 320 abstracts, 76 were excluded because they were not original research studies, thus reducing the number of potentially eligible articles to 244. Of the 244 abstracts, 89 abstracts were excluded because they did not include a human population. As a result, 155 articles were sought for retrieval, of which 13 were not found. The 142 articles for which a full-text PDF was retrieved were screened for inclusion criteria 4 and 5, related to the study population and investigation of the metabolic phenotype, respectively. Ninety-seven studies did not meet the population criterion, the metabolic phenotyping criterion, or both. Furthermore, 12 studies were retrospectively excluded despite meeting the inclusion criteria because they did not analyse statistical differences between Long COVID patients and healthy controls or recovered individuals (*n* = 10)*,* because the only metabolite analysed did not differ between patients and controls (*n* = 1), or because only lipids were analysed (*n* = 1), thus yielding a total of 33 studies eligible for inclusion in the review.

**Fig. 1:**
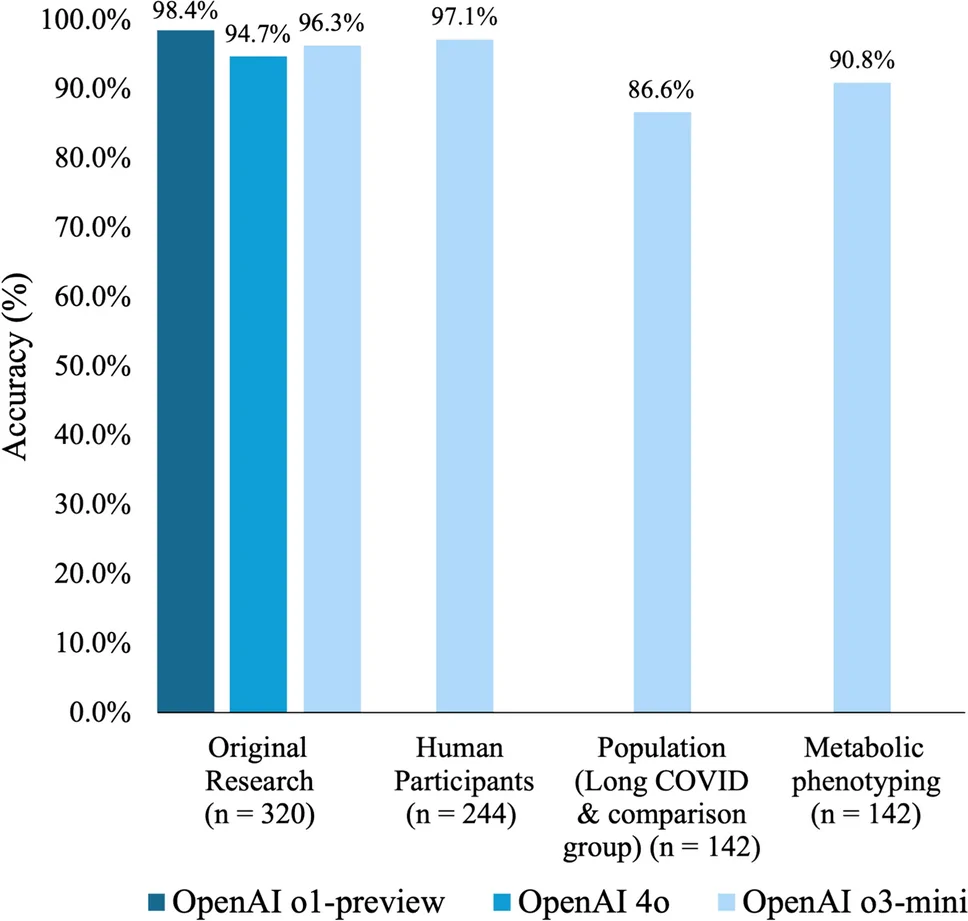
Accuracy of AI in screening articles for inclusion criteria compared to gold standard

**Fig. 2:**
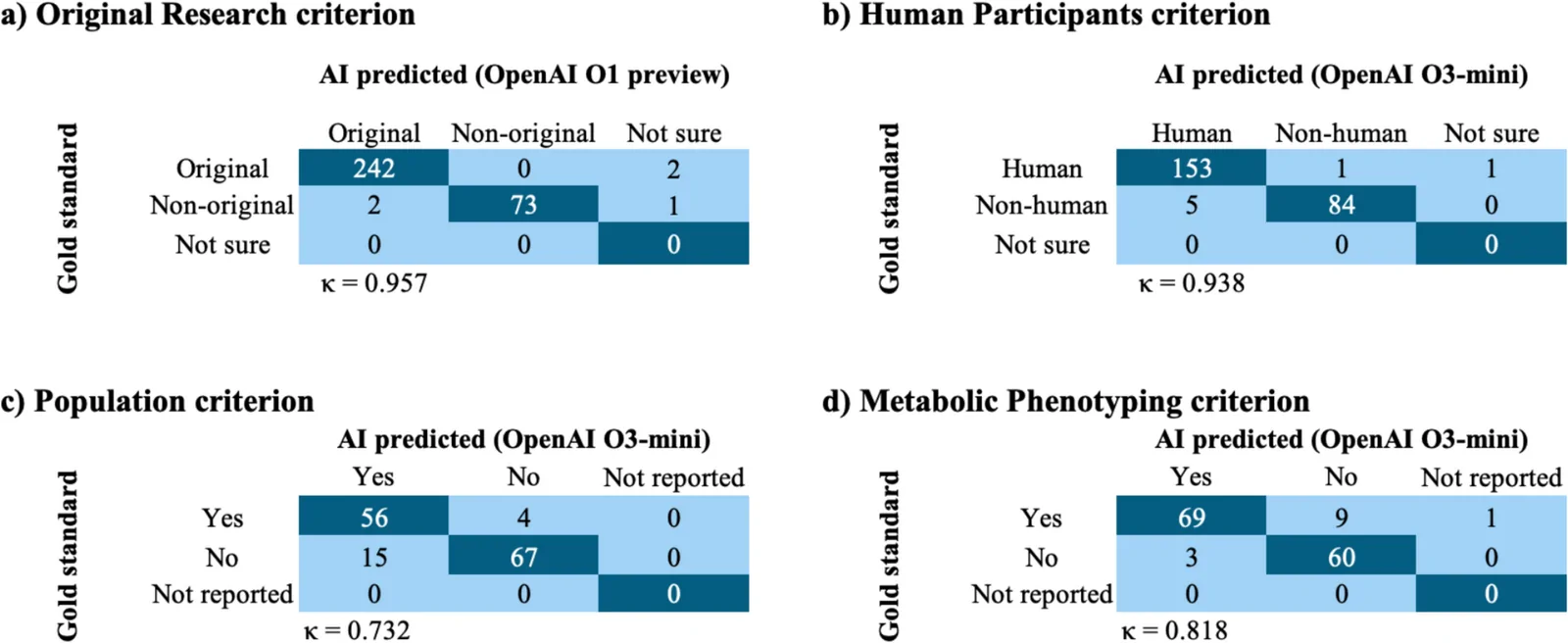
Confusion matrices indicate the agreement between AI and the gold standards in evaluating inclusion criteria. Rows indicate the composition of the manually created gold standard categories, while columns indicate how predicted categories were composed. Values along the diagonal (highlighted in dark blue) indicate the number of articles for which there was agreement between the gold standards and the responses generated by AI

The accuracy of OpenAI's GPT-4o model in identifying whether abstracts corresponded to original investigations or not was 94.7% and increased further to 96.3% and 98.4% when queried using the o3-mini and o1-preview models, respectively (Fig. 1).The model o1-preview correctly identified 242 out of 244 abstracts meeting the originality criterion and accurately recognised 73 out of 76 non-original abstracts, yielding an "almost perfect agreement" (κ = 0.957) according to the Landis & Koch (1977) evaluation (Fig. 2a), and F1 scores of 99.6% and 98.6% for "Yes" and "No" responses, respectively (Table 2). There were two false positives and three instances where AI responded with “Not sure”. Out of the five instances where AI provided an incorrect response, one was due to the study not having an abstract; and as a result, there was no input data for AI to evaluate (Comellas and Fain 2024). In one instance where AI incorrectly returned "Yes", the study was a review article, but this was not clear from the abstract (Guarnieri et al. 2024). Since AI only had access to the abstract to determine whether or not the study was an original investigation, it did not have sufficient information to generate a correct response. Based on the accuracy, cost, and time investment to complete the screening for the Original Research criterion using the three different OpenAI models (Table 3), it was decided to complete all subsequent steps with model o3-mini.

**Table 2:** Agreement metrics between AI and gold standard per inclusion criterion

Criterion	Precision	Negative predictive value	Sensitivity	Specificity	F1		Support	
					Yes	No	Yes	No
Original research	99.2%	100%	100%	97.3%	99.6%	98.6%	242	75
Human	96.8%	98.8%	99.4%	94.4%	98.1%	96.6%	154	89
Participants	78.9%	94.4%	93.3%	81.7%	85.5%	87.6%	60	82
Metabolic phenotyping	95.8%	87.0%	88.5%	95.2%	92.0%	90.9%	78	63
Mean	92.7%	95.1%	95.3%	92.1%	93.8%	93.4%		
SD	9.3%	5.9%	5.4%	7.1%	6.4%	5.1%		

AI results were obtained with model o1-preview for the original criterion and with model o3-mini for all remaining criteria

**Table 3:** Time and costs associated with AI screening articles for inclusion criteria

Prompt	Original research			Human participants	Population & metabolic phenotyping
Model	OpenAI model GPT-4o	OpenAI model o3-mini	OpenAI model o1-preview	OpenAI model o3-mini	OpenAI model o3-mini
Number of abstracts or articles	320	320	320	244	142
Time (min)	36	44	95	32	102
Cost (USD)	< 2	0.52	33.70	0.33	4

In identifying whether abstracts regarded studies investigating human participants, the OpenAI o3-mini model yielded an accuracy of 97.1% (Fig. 1). One hundred fifty-three out of the 155 abstracts were correctly labelled as research with human populations, while 84 out of 89 were correctly classified as non-human research (Fig. 2b). This corresponded to F1 scores of 98.1% and 96.6% for “Yes” and “No” responses, respectively (Table 2), and nearly perfect inter-rater agreement (κ = 0.938) (Fig. 2b). Three out of the seven mistakes AI made on this criterion regarded protocol studies, i.e., articles describing detailed protocols for yet-to-be-conducted research on human participants without executing the protocol and presenting the results (Hanna et al. 2020; Busatto et al. 2021; Jegal et al. 2022). Furthermore, one study did not describe the investigation of humans in the abstract, and AI did therefore not have the information required to provide the correct response based on the abstract alone (Wong et al. 2023). For the Original Research and Human Participants prompts, the average number of input tokens was approximately 600 per abstract, while the average number of output tokens was approximately 180 per answer.

Of the 142 articles screened for the inclusion of Long COVID patients and a comparison group, 123 were correctly classified into "Yes" (*n* = 56) and "No" (*n* = 67), yielding substantial agreement with the gold standard (accuracy 86.6%, κ = 0.732, Fig. 1 & Fig. 2c). Precision, negative predictive value, sensitivity, and specificity were 78.9%, 94.4%, 93.3%, and 81.7%, respectively (Table 2). AI identified the use of metabolic phenotyping methods with an accuracy of 90.8% (Fig. 1). Sixty-nine out of the 79 studies using metabolic phenotyping approaches were correctly recognised (precision = 95.8%, sensitivity = 88.5%), and 60 out of the 63 non-metabolomics articles were accurately responded to with "No" (negative predictive value = 87.0%, specificity = 95.2%) (Table 2). The agreement between AI and the gold standard for this criterion was "almost perfect" (κ = 0.818). Input and output tokens were on average ~ 20,456 and ~ 214 per article. The mean accuracy, precision, negative predictive value, sensitivity, and specificity of AI across all inclusion criteria were 93.3% ± 5.5% (86.6%-98.4%), 92.7% ± 9.3% (78.9%-99.2%), 95.1% ± 5.9% (87.0%-100%), 95.3% ± 5.4% (88.5%-100%), and 92.1% ± 7.1% (81.7%-97.3%), respectively, considering responses obtained with OpenAI's o1-preview model for the original research criterion and OpenAI’s o3-mini model for all other inclusion criteria (Table 2). AI completed the screening stage in less than three hours, at a cost of approximately 4.85 USD (considering the time and cost of running the screening steps with OpenAI’s o3-mini model; Table 3). The time required to manually complete these screening steps in duplicate was ~ 45 h, equivalent to ~ 745 USD at the Australian minimum hourly wage.

### Risk of Bias Assessment

**Fig. 3:**
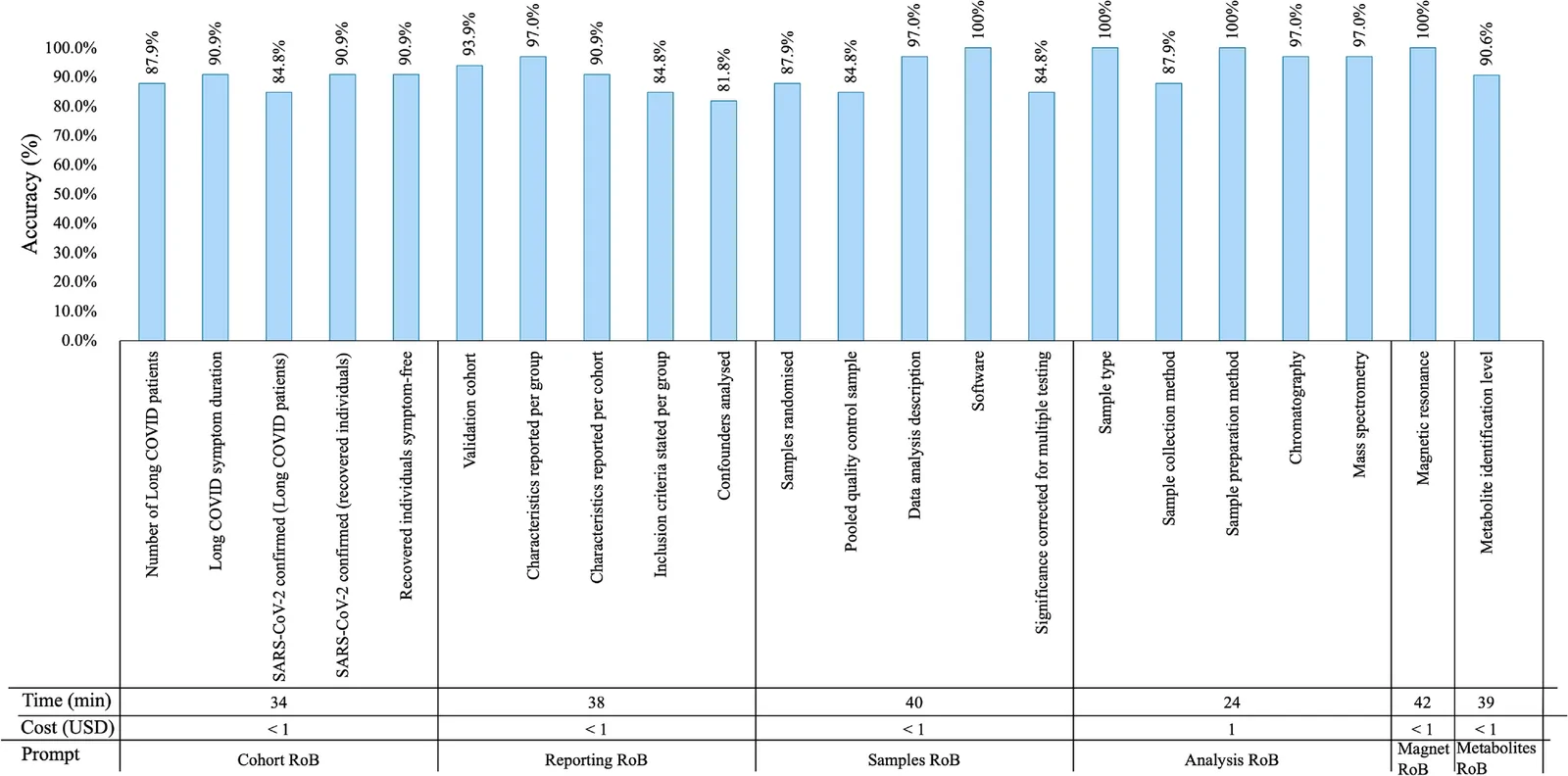
Accuracy of risk of bias assessment performed by AI versus the gold standard, with associated time and costs

The accuracy of AI in assessing the RoB of the 33 included articles across 22 pre-defined criteria ranged between 81.8% and 100%, with a mean accuracy of 91.9% ± 5.9% (Fig. 3). Confusion matrices detailing the agreement between the manually created gold standard RoB assessment and the assessment generated by AI (including Cohen's κ), specifying the composition of the gold standard and predicted categories, are provided per RoB criterion in Supplementary Fig S1. AI performed the RoB assessment in approximately three hours and forty minutes, while manual completion of this task in duplicate took ~ 40 h. The average number of input tokens ranged between ~ 19,964 and ~ 22,668, while output tokens ranged from ~ 126 to ~ 484, per article and per RoB prompt. The costs for running these RoB prompts on OpenAI's o3-mini model for 33 articles were less than 6 USD, compared to ~ 650 USD for manual assessment.

### Data Extraction

**Fig. 4:**
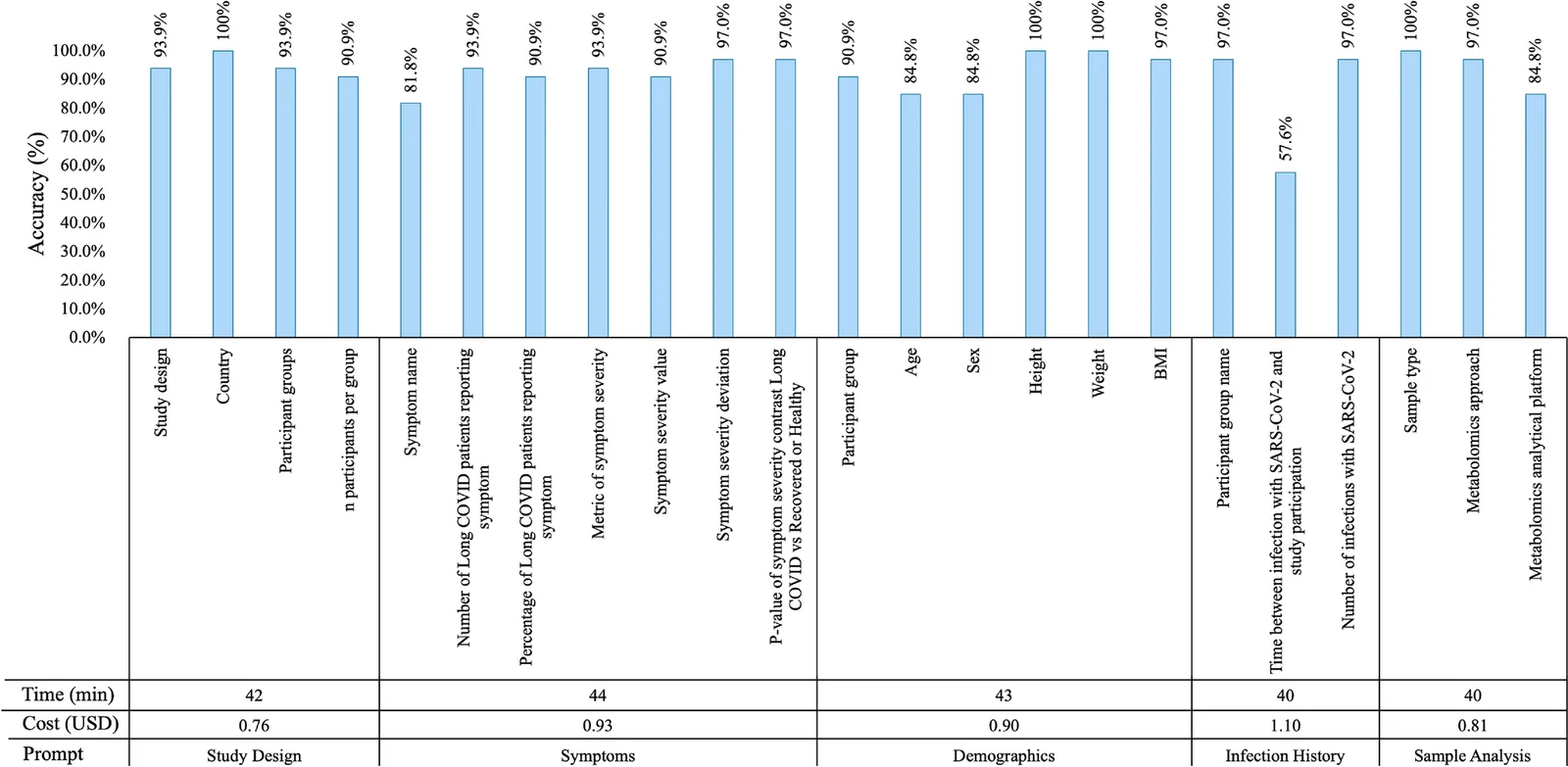
Accuracy of data extraction performed by AI versus the gold standard, with associated time and costs

AI extracted 23 parameters from the included studies with a mean accuracy of 92.0% ± 9.2% (57.6%-100%). The average accuracy in the study design, symptoms, demographics, infection history, and sample analysis domains was 94.7% ± 0.04%, 92.2% ± 0.05%, 92.9% ± 0.07%, 83.8% ± 0.23%, and 93.9% ± 0.08%, respectively (Fig. 4). Notably, AI did not make any mistakes in extracting the countries in which each study was conducted and the heights and weights of all participants. The lowest accuracy (i.e., 57.6%) was obtained for extracting the time that had elapsed between infection with the SARS-CoV-2 virus and participation in the study. Manually extracting data from the included articles was completed in duplicate in ~ 32 h, while AI completed this stage in approximately three and a half hours. Input tokens varied from ~ 19,612 to ~ 27,306, while output tokens ranged between ~ 163 and ~ 626, per article and per Data Extraction prompt. Associated costs of using AI were about 4.50 USD, while the minimum wage equivalent of the human work was ~ 520 USD.

In screening articles for eligibility, performing RoB assessment, and extracting data, AI made a total of 164 mistakes. These mistakes were categorised using a multidimensional approach, and the results are presented in Table 4. Several errors did not fit into merely one class and were therefore categorised into multiple classes. The total number of mistakes per category in order from most to least common was as follows: information omission error (*n* = 83), comprehension error (*n* = 49), instruction-following error (*n* = 22), hallucination error (*n* = 14), inference error category b: failure to make correct implicit inferences (*n* = 12), source-related error (*n* = 11), inference error category a: incorrect inference drawn from the text beyond what was explicitly stated (*n* = 7), irrelevance error (*n* = 1) (Table 4).

**Table 4:** Multidimensional evaluation of the mistakes AI made categorised per prompt and criterion

Prompt (AI model):	Criterion:	Total number of mistakes	Comprehension error	Information omission error	Hallucination error	Inference error		Instruction-following error	Irrelevance error	Source-related error
Original Research (OpenAI o1-preview)	Original	5								5
Human Participants (OpenAI o3 mini)	Human	7	5	3				1		3
Inclusion Criteria (OpenAI o3-mini)	Population	19	15	3	1			4		
	Metabolic phenotyping	13	8	1	1	1		1		2
Cohort RoB (OpenAI o3-mini)	Long COVID number	4			2		1	2		
	Symptom duration	3		3			2			
	Infection	5		1	3	1	1			
	Definition recovered	3		2	1	1		1		
	Recovered	3		1			2			
Reporting RoB (OpenAI o3-mini)	Validation cohort	2	2	1						
	Characteristics reported	1			1					
	Characteristics per cohort	3	2	2						
	Inclusion criteria	5	2	2				2	1	
	Confounders	6	3	4				2		
Samples RoB (OpenAI o3-mini)	Randomisation	4	3	1				3		
	Quality control	5	1			2		3		
	Data analysis	1	1							
	Software	0								
	Significance level	5	3		2					
AnalysisRob (OpenAI o3-mini)	Sample type	0								
	Sample collection	4		2	1	1				
	Sample preparation	0								
	Separation (chromatography)	1								1
	Mass spectrometry	1						1		
MagnetRob (OpenAI o3-mini)	Magnet	0								
MetabolitesRob (OpenAI o3-mini)	Metabolite identification level	3	3	2	1					
Design (OpenAI o3-mini)	Study design	2					2			
	Country	0								
	Participant groups	2		2						
	Number of participants per group	3		3						
Symptoms (OpenAI o3-mini)	Name	6		6						
	*n* reported by	2		2						
	% reported by	3		3						
	metric	2		2						
	value	3		2				1		
	deviation	1		1						
	p-value	1		1						
Demographics (OpenAI o3-mini)	Name	3		3						
	Age	5		5						
	Sex	5		5				1		
	Height	0								
	Weight	0								
	BMI	1		1						
Infection history (OpenAI o3-mini)	Name	1		1						
	Time since infection	14		13	1	1	3			
	Number of infections	1					1			
Sample analysis (OpenAI o3-mini)	Sample	0								
	Metabolomics	1	1							
	Analytical platform	5		5						
	**Total:**	**164**	**49**	**83**	**14**	**7**	**12**	**22**	**1**	**11**

Errors may be categorised into more than one category

## Discussion

This study presents the first comprehensive evaluation of AI performance across all three critical stages of systematic literature reviews in metabolomics research: article screening, RoB assessment according to established metabolomics quality assessment guidelines (Sumner et al. 2007; U et al. 2022), and data extraction. Our AI-assisted workflow achieved mean accuracies of 92.7% ± 4.9%, 91.9% ± 5.9%, and 92.0% ± 9.2% across these stages when contrasted against manually obtained gold standards, respectively, while reducing processing time from ~ 116 h to ~ 10 h and reducing costs from approximately 2000 USD (Australian minimum wage equivalent) to 15.35 USD. These findings demonstrate the potential for AI to significantly accelerate systematic literature reviews in metabolomics while maintaining acceptable accuracy levels.

The screening accuracy results obtained in this study demonstrate substantial improvements over previously reported performance metrics. Compared to the study by López-Pineda et al. (2025), who used OpenAI model GPT-4o mini for screening titles and abstracts, we found higher sensitivity (95.3%) but similar specificity (92.1% vs 95.1%) using model o3-mini to screen abstracts for our inclusion criteria. The discrepancy in sensitivity may partially be due to the different LLMs employed. OpenAI's o3-mini model was developed to excel at complex reasoning tasks, while model GPT-4o mini performs better at focused tasks (OpenAI 2025c). Indeed, using model GPT-4o, we found lower accuracies in screening abstracts as compared to the o3-mini and o1-preview models. AI gave 14 false-negative responses across all inclusion criteria. Of these, four articles were manually deemed eligible for inclusion, emphasising the importance of human oversight.

For the RoB assessment, the results exceeded those reported by Eisele-Metzger et al. (2025), who investigated the performance of the AI model Claude 2 in evaluating the RoB of 100 RCTs using four RoB criteria. They found agreements ranging between 58% and 71% on the four criteria (Eisele-Metzger et al. 2025), which were considerably lower than the mean accuracy of 91.9% ± 5.9% (81.8%-100%) that we obtained across the 22 RoB criteria against which our 33 articles were evaluated. Eisele-Metzger and colleagues (2025) concluded that Claude should not be utilised as a stand-alone method to perform a RoB assessment. In addition to the difference in AI models used, the RoB assessment tools, criteria, and study designs of the articles under evaluation were also very different between the study by Eisele-Metzger et al. (2025) and the present study, and may be responsible for the discrepancy in AI accuracies. Eisele-Metzger et al. (2025) used the Cochrane RoB 2 tool, while we used a modified version of a previously published tool to assess metabolomics research (U et al. 2022), and they specifically evaluated RCTs, wheras our review did not include any RCTs.

It must be noted that some of the cited research used larger numbers of abstracts and articles than we did in the present study (e.g., 984 vs 320 abstracts (López-Pineda et al. 2025) and 100 vs 33 articles (Eisele-Metzger et al. 2025), and further research is required to verify the comparatively high accuracies obtained in this study on larger volumes of literature.

The data extraction performance (92.0% mean accuracy) represents a notable advance over previous expectations. Wagner expected "moderate potential" for qualitative data extraction (Wagner et al. 2022), and Blaizot and colleagues (Blaizot et al. 2022) concluded in their systematic literature review that using AI to extract data for evidence synthesis remained challenging and required human intervention to finalise the data extraction process. The recommendations from these past studies mainly regarded the use of AI-based software tools such as Rayyan (Ouzzani et al. 2016), RobotReviewer, Abstrackr, and EPPI-Reviewer 4 which assisted with screening articles or extracting data from articles, while we took a different approach and used an LLM that was queried with highly specific, human-written prompts. Our approach yielded accuracies high enough to minimise human intervention to mere verification of AI's output.

Across the three stages that were evaluated in this study (i.e., screening for inclusion criteria, RoB assessment, and data extraction), AI yielded a mean accuracy of 92.0% ± 7.5% in comparison to the manually computed reference standards. With the exception of one data extraction parameter, AI completed all tasks with accuracies above 80%. The exception regards the extraction of the time window between participants' infection with the SARS-CoV-2 virus and participation in the study (Infection History Prompt – Time between infection with SARS-CoV-2 and participation, Fig. 4), for which AI only yielded an accuracy of 57.6%. Most of these errors were classified as information omission errors, indicating that AI omitted information in the articles that was relevant to answer the criterion correctly. A potential explanation for this observation could be the diversity in the way in which this information was reported across different studies. For example, one study described the relevant variable as "time since acute infection (d)" (Mikuteit et al. 2023), while other studies described this as "time after exposure [months]" (Kovarik et al. 2023), "days after infection sampled" (Guo et al. 2023), or "time from COVID-19 diagnosis" (Tosato et al. 2022). The manner of describing the information was different in the article as compared to how it was described in the prompt, which may have made finding the relevant information particularly challenging for AI.

Systematic evaluation of the errors made by AI in this study revealed that the majority of errors (i.e., 83 out of 164, 50.6%) were due to AI omitting information provided in the source (i.e., the abstract or article). Various factors may underlie this observation. As explained in the paragraph above, diversity in phrasing similar information between different articles could be one possible factor,the reporting of findings in graphs and figures could be another. OpenAI's o3-mini model was used in this study, which was not trained to extract data from images. More recently published AI models have been trained to do so and using these models may improve the accuracy of AI in extracting data from articles. In 49 instances (29.9%), AI misinterpreted the task specified in the prompt or information provided in the article, while in 11 instances (6.7%), the information provided in the source was absent or ambiguous and made it difficult even for human reviewers to correctly answer the criterion. More explicitly phrasing methods and results in scientific papers may enable AI to overcome some of these errors. AI made a total of 19 inferencing errors, drawing incorrect inferences from the text beyond what was explicitly stated seven times, and failing to make correct implicit inferences in 12 instances. Together with the 14 times that AI hallucinated, inventing data that was not reported in the source, these errors emphasise once more the responsibility of human researchers to oversee the process and verify the results generated by AI.

Systematic literature reviews are of great benefit to the metabolomics field, as they enable the validation of metabolic biomarkers across published cohorts. By comprehensively evaluating methods and synthesising results, they enhance the reliability of conclusions and have the potential to accelerate the translation of metabolomics findings into clinical applications. The currently proposed workflow reduced the time investment for a systematic literature review of the Long COVID metabolomics literature from ~ 116 h to ~ 10 h (excluding the time required for human reviewers to evaluate AI’s responses). This demonstrates its potential to considerably reduce the time and burden of conducting systematic literature reviews, making such reviews more achievable for researchers and resulting in possible associated benefits for clinical practice. Although this study illustrated the hybrid human-AI workflow for systematic literature reviews using a relatively small set of articles on a niche topic and using a domain-specific RoB tool, the general workflow is domain-agnostic and could be adapted to other fields.

### Limitations

Although we found generally good agreement between OpenAI model o3-mini and manually created gold standards, without any systematic errors, the failure of AI to achieve accuracies of 100% across all steps emphasises a need for human involvement. Based on our results, we suggest a methodological framework that integrates AI in the traditional systematic literature workflow. This workflow, which is described in more detail in the section "Recommendations for the Implementation of AI in Conducting Systematic Literature Reviews", relies on humans to retrieve literature and limits the role of AI to that of a first reviewer in screening for inclusion criteria, RoB assessment, and data extraction, the results of which must always be inspected and corrected where necessary by a human investigator (Haman and Školník 2023). Nonetheless, AI is evolving extremely rapidly and future models may be able to complete additional tasks and further improve accuracy. This study focused only on the metabolomics literature of a single medical condition and, after testing several models, evaluated the performance of one AI model. Based on our results, we argue that the hybrid human-AI workflow for systematic literature reviews may be adapted to other areas; however, generalisability to other topics and AI models requires further investigation. Additionally, based on the results obtained with OpenAI's o3-mini model in the present study, we emphasise the importance of having human researchers verify all of AI's responses; however, AI models may evolve to a stage where some tasks can be labelled as low-risk, and may subsequently not require human verification anymore.

Another limitation of this workflow lies in the inconsistency of AI's responses. LLMs are stochastic (i.e., random) token generators, with each token being sampled from a probability distribution. This indicates that querying the same prompt multiple times can result in different – but valid – responses each time (Holtzman et al. 2019). Using OpenAI's API, the non-deterministic behaviour, and thus the degree of consistency, can be improved by adjusting parameters such as temperature, seed, and top_p. In the present study, we retained the default settings for these parameters and controlled variability by constraining the output format through the prompt. Specifically, the prompt instructions embedded a JavaScript Object Notation (JSON) structure (keys, types, allowed values) and required the AI model to return only that JSON. This standardised structure not only enabled systematic parsing and seamless merging of AI's responses, it also constrained AI’s possibilities in generating a response. Conceptually, this approach aligns with OpenAI's Structured Outputs guidance on schema-adherent generations, although the constraint was enforced through the prompt rather than through parameter settings (OpenAI API 2025d, e). A practical limitation of the currently presented workflow is that querying commercial AI services for the screening of hundreds, or even thousands, of articles through the API requires programming skills. This method may therefore be impractical for many researchers and emphasises the need for a user-friendly interface where researchers can upload their abstracts or PDF articles of interest, write prompts, and query AI via the API (López-Pineda et al. 2025).

Although the direct financial cost of using AI in this study was minimal (i.e., ~ 15.35 USD), it must be noted that intensive AI usage also incurs an energy cost. This energy demand is often met by fossil fuels, which contribute to carbon dioxide and other greenhouse gas emissions (Budennyy et al. 2022; Masciari and Napolitano 2025). Indeed, recent analyses showed that the training, inference, and management of LLMs in data centres drive incremental energy demand and that full lifecycle assessments (including hardware manufacture, cooling systems, and energy mix) are needed to characterise the true environmental burden (Masciari and Napolitano 2025). As interest in AI applications continues to grow, it is essential that sustainability issues are addressed through research and innovation. This includes innovations in the computational research domain aimed at improving algorithmic efficiency to reduce the energy consumption involved in training and deploying AI, as well as advancements in the usage of renewable energy sources (Budennyy et al. 2022).

Finally, using AI in research raises complex ethical challenges, particularly around authorship, transparency, accountability, and integrity (Resnik and Hosseini 2024; Bjelobaba et al. 2025). The status quo is that AI should not be listed as authors because it cannot assume responsibility for the accuracy, originality, or integrity of the work. Accountability resides with the human researchers, emphasising the importance of critically reviewing the work generated by AI and the ethical imperative of complete transparency in disclosing AI's role. Previous studies have provided further recommendations for ethical use of AI in research (Resnik and Hosseini 2024; Bjelobaba et al. 2025).

### Recommendations for the Implementation of AI in Conducting Systematic Literature Reviews

**Fig. 5:**
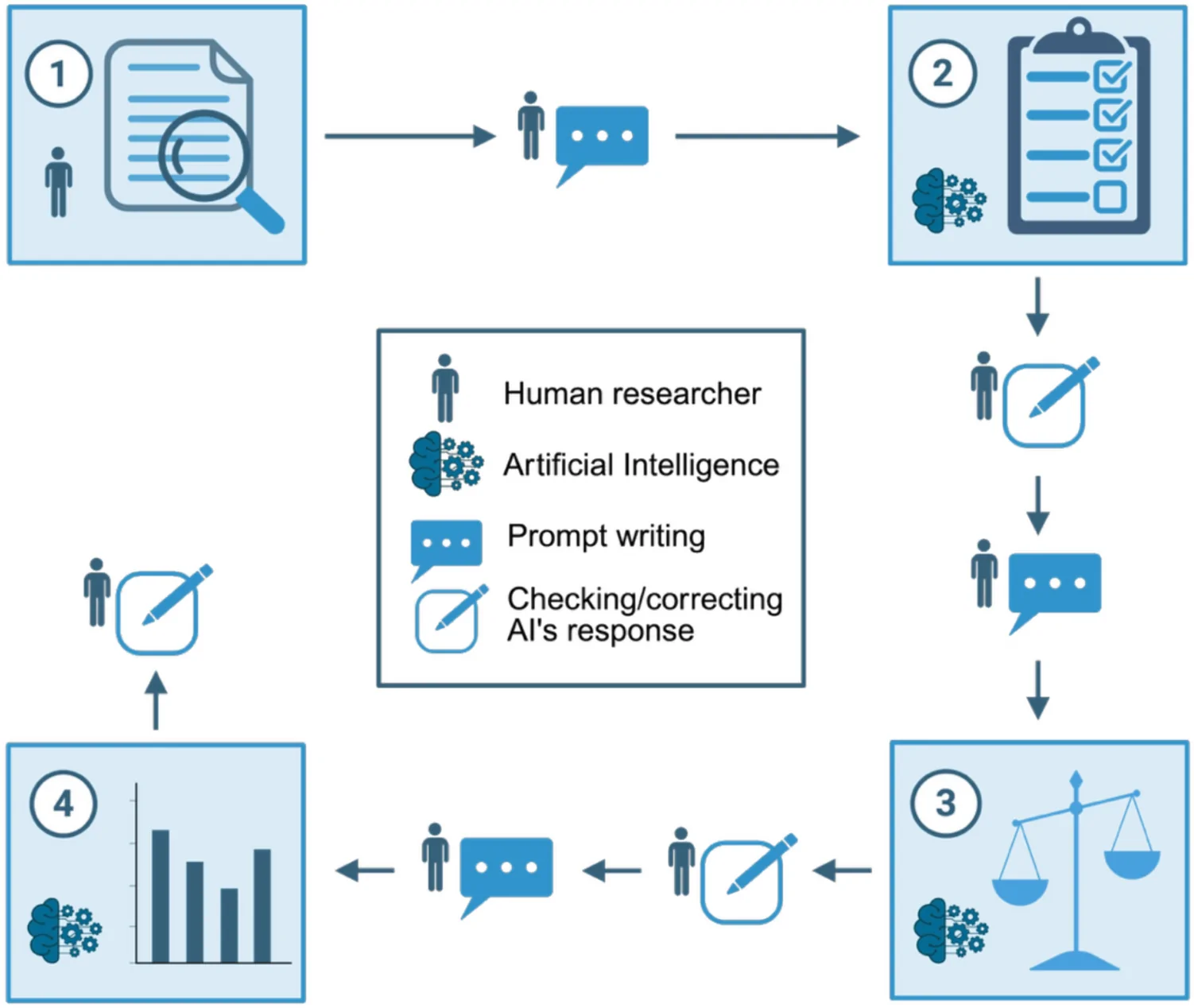
Schematic overview of the proposed hybrid human-AI workflow for systematic literature reviews. The workflow illustrates alternating roles of the human researcher and AI. The human researcher initiates the process by searching the literature (step 1) and writing prompts to query AI for article screening. AI screens articles for inclusion criteria (step 2) and returns results, which are subsequently verified by the human researcher. The human researcher writes prompts to query AI to complete the RoB assessment, which AI performs in step 3. Similarly to previous steps, AI's results are verified and corrected where necessary by the human researcher, who subsequently writes prompts to query AI for data extraction. Finally, AI completes data extraction (step 4), and the results are again checked and modified by the human researcher. In this proposed workflow, AI performs the largest volume of the work, limiting the role of the human researcher to writing prompts and checking AI’s output. Figure created with BioRender

**Fig. 6:**
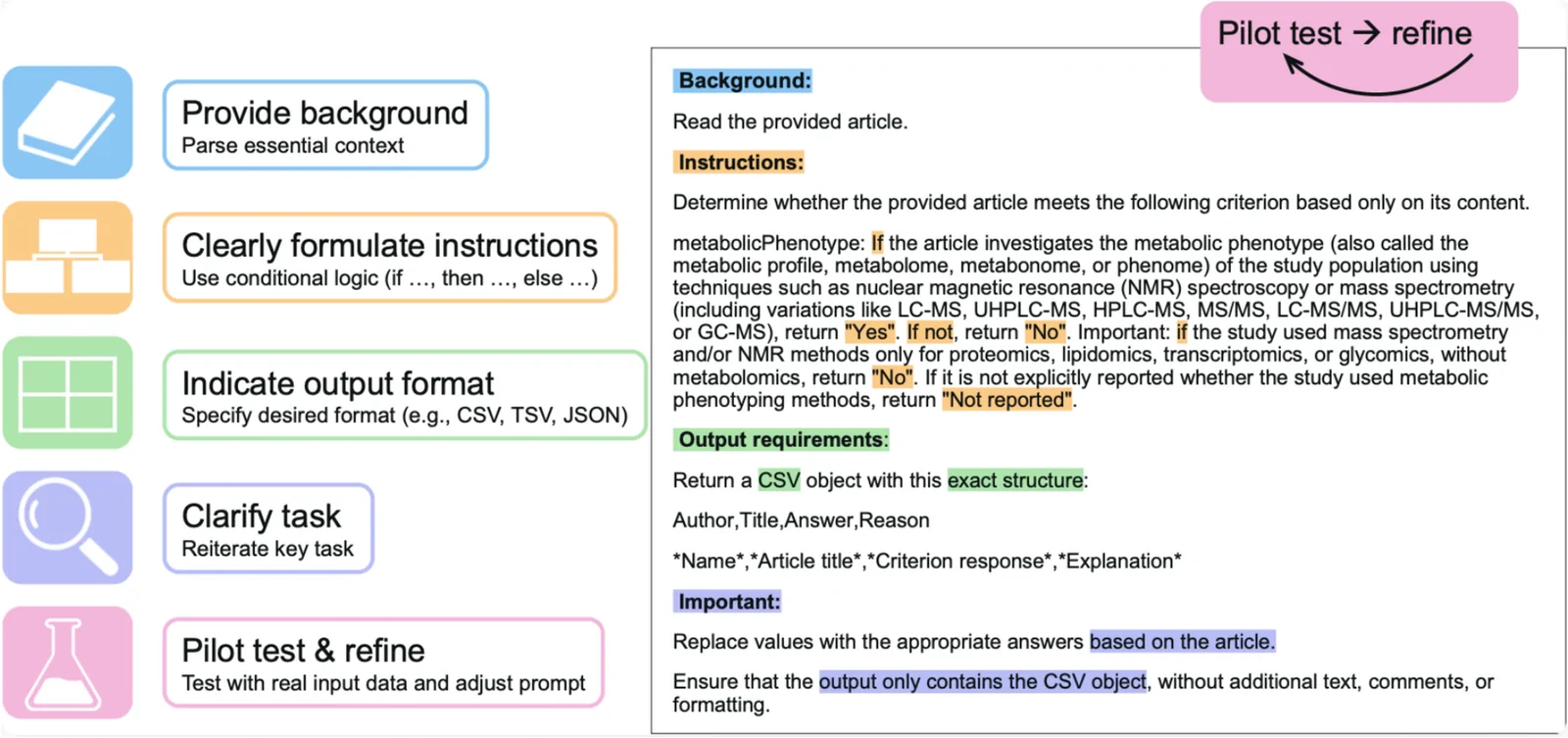
Main components of an exemplar AI prompt that may be used in literature screening. The left panel summarises five key prompt design principles, while the right panel shows an example prompt with corresponding highlights indicating how each principle is implemented

Although this study illustrated the implementation of AI in conducting a systematic literature review using metabolomics literature, the proposed workflow is not restricted to metabolomics and may be adapted to any non-metabolomics field. In the following, we provide a proposal for how future studies may integrate AI into the systematic literature review workflow. First, based on the results of this study, we suggest that AI might work alongside human researchers in completing systematic literature reviews (Fig. 5). In this workflow, human researchers retrieve literature of interest, with possible AI assistance in developing search strings for online databases such as PubMed and Scopus. AI can then be prompted to obtain preliminary results regarding inclusion criteria, RoB assessment, and data extraction, followed by the verification and, if necessary, the correction of results by a human researcher (Fig. 5). Second, in line with previous findings, we found that there is no room for ambiguity when designing prompts for AI (Alshami et al. 2023). Prompts must be engineered very clearly and systematically, and they require extensive testing and optimisation before yielding desirable outputs. If-else guidance in the prompt proved highly valuable given the categorical nature of the inclusion criteria and RoB prompts. By specifying which response should be provided if a study did not use the method under evaluation (e.g., "If the study did not use chromatography to analyse Long COVID samples for metabolomics purposes, return "NA" and proceed with answering the next question", Analysis RoB Prompt-Separation criterion) or if certain information was explicitly reported in an article, and what to do if it was not (e.g., "If the study reports which biological sample types were collected from Long COVID patients for metabolomics analysis, return "1". If not explicitly reported, return "-1".", Analysis RoB Prompt-SampleType criterion), AI was guided through the answer possibilities. We provide a generic prompt template that authors may tailor to their own inclusion criteria, RoB tool (e.g., Risk Of Bias In Non-randomized Studies—of Interventions (ROBINS-I) or version 2 of the Cochrane RoB tool for randomized trials (ROB-2) (Sterne et al. 2016, 2019)), and data extraction parameters (Fig. 6). The key components of this prompt template are background information, clearly formulated instructions, an indicator of the output format, and clarification of the task (Fig. 6). All prompts used in the present study followed this template and resulted in only one AI irrelevance error, i.e., where AI did not address all aspects of the prompt and user intent (Table 4). Researchers who adopt the proposed template are encouraged to conduct preliminary pilot tests on their prompts, and to utilise AI’s responses as a guide to improve the prompts’ clarity. Additionally, researchers are encouraged to pilot test various contemporary AI models and to implement the one with the best performance.

Researchers adopting the currently proposed workflow using the API from OpenAI may consider adjusting the seed and temperature or the seed and top_p settings (note that OpenAI advises against adjusting both temperature and top_p simultaneously) in addition to standardising the output format, with the aim of obtaining less random, more deterministic responses. Specific recommendations are provided in OpenAI's API reference (OpenAI API 2025d, e).

### Recommendations for the Reporting of Methods

Ambiguity or lack of detail in the input data has detrimental effects on the accuracy of AI. Here, the input data consisted of abstracts and full-text articles, emphasising the importance of clearly written research papers that describe the study population and analytical methods in sufficient detail, instead of merely citing previously published work. In line with the Strengthening the Reporting of Observational Studies in Epidemiology (STROBE) guidelines for observational studies and the CONsolidated Standards Of Reporting Trials (CONSORT) checklist for randomised trials (Cuschieri 2019; Hopewell et al. 2025), we advise authors to report at least the following parameters in their methods: in- and exclusion criteria and number of participants per group, statistical software and tests, confounder analysis, and *p*-value correction method. For metabolomics research, we suggest authors minimally report items provided in the "Chemical Analysis" and "Metabolite Identification" domains of the RoB tool proposed in this study, which incorporate items from a previously published RoB tool, the MSI, and the MQACC (Sumner et al. 2007; U et al. 2022; Kirwan et al. 2022). For research in other areas, authors may consider established tools such as ROB-2 and ROBINS-I for randomised trials and non-randomised interventions, respectively, as guidelines for minimum reporting (Sterne et al. 2016, 2019).

## Conclusion

We propose a novel workflow for the implementation of AI in the systematic literature review process. We investigated the performance of AI in screening abstracts for four inclusion criteria, evaluating the quality of articles against 22 RoB criteria, and extracting 23 parameters including descriptive, demographic, and analytical details, and found a mean accuracy of 92.0% ± 7.5% across these stages. AI performed these steps in circa 10 h, while it took ~ 116 h for human reviewers to complete the process in duplicate. Associated costs of running this workflow with OpenAI's o3-mini model were approximately 15.35 USD (approximately 0.45 USD per article) compared to ~ 2000 USD (~ 60.6 USD per article) for completing the workflow manually at minimum wage. These results indicate the potential of AI to considerably reduce the time required to conduct systematic reviews of the literature, at minimal cost and with limited need for human interference. Nonetheless, care should be taken in manually verifying AI's responses to ensure accuracy. We suggest an innovative hybrid AI-human workflow, where human researchers search and retrieve literature from online databases, followed by the independent completion of inclusion criteria screening, RoB assessment, and data extraction by AI, with a human researcher verifying and, where necessary, correcting AI’s preliminary results. For the RoB assessment, we developed a novel tool tailored to metabolomics studies based on well-established quality criteria. This tool may be of interest to journals in the metabolomics domain as a guideline for minimum reporting standards, with benefits for reproducibility and accuracy of metabolite identification. Importantly, we here applied the integration of AI in the systematic literature review workflow using metabolic phenotyping studies into Long COVID, but the method is generically adaptable to other research fields using the proposed workflow and prompt template. Future research should concentrate on investigating AI's accuracy in completing steps of the currently proposed workflow applied to other topics, testing whether novel AI models are able to complete relevant tasks error-free (with a potential for low-risk tasks to be completed independently without human verification), solving ethical considerations associated with the use of AI in research, advancing innovations to improve sustainability and reduce the carbon footprint associated with intensive AI use, and creating user-friendly software for AI-assisted evidence synthesis. In conclusion, results of this study indicate that human researchers and AI may jointly collaborate in completing systematic literature reviews, although caution is warranted in generalising the proposed workflow to other research domains and other AI models, as well as handling ethical and environmental concerns related to intensive AI usage.

## Supplementary Information

Below is the link to the electronic supplementary material.

Supplementary file 1.

## Data Availability

The prompts and search strings are made available in the Supplementary File. The raw output data from AI and the reference data created by human reviewers are deposited in the Open Science Framework (OSF) repository (https://doi.org/10.17605/OSF.IO/H9P84). An overview of the mistakes made by AI in comparison to the manually extracted results for each prompt is also provided in the OSF repository, as is a logbook of the work conducted by the human reviewers to complete screening, RoB assessment, and data extraction.

## Abbreviations

AI: Artificial intelligence
API: Application programming interface
BMI: Body Mass Index
CONSORT: CONsolidated Standards Of Reporting Trials
COVID: Coronavirus disease 2019
JSON: JavaScript Object Notation
LLM: Large language model
MQACC: Metabolomics Quality Assurance and Quality Control Consortium
MRI: Magnetic resonance imaging
MRS: Magnetic resonance spectroscopy
MSI: Metabolomics Standards Initiative
NMR: Nuclear magnetic resonance
OSF: Open Science Framework
PRISMA: Preferred Reporting Items for Systematic Reviews and Meta-Analyses
RCT: Randomised controlled trial
RoB: Risk of bias
ROBINS-I: Risk Of Bias In Non-randomized Studies of Interventions
ROB-2: Version 2 of the Cochrane risk-of-bias tool for randomized trials
SARS-CoV-2: Severe acute respiratory syndrome coronavirus 2
STROBE: Strengthening the Reporting of Observational Studies in Epidemiology
