# Supplementary material for: Artificial Intelligence-Assisted Systematic Literature Reviews in Metabolic Phenotyping: A Methodological Framework and Validation Study
Source: Phenomics. 2026 Aug 17;6(4):372–90. doi: 10.1007/s43657-026-00336-y (PMC13713925; doi:10.1007/s43657-026-00336-y)
Supplement: Supplementary file 1 [file 43657_2026_336_MOESM1_ESM.docx]

SUPPLEMENTARY FILE FOR:

**Artificial Intelligence-Assisted Systematic Literature Reviews in Metabolic Phenotyping: A Methodological Framework and Validation Study**

**Maartje Cox** (ORCID 0009-0003-7870-9183)^1^*, Javier Osorio Mosquera (ORCID 0000-0002-8278-3103)^1,2^, Shaimaa Khandaker^1^, Samuele Sala^1^, Julien Wist (ORCID 0000-0002-3416-2572)^1,2,3^, Jeremy K Nicholson (ORCID 0000-0002-8123-8349)^4^, Elaine Holmes^1,3,4^, Timothy Fairchild (ORCID 0000-0002-3975-2213)^5,6^, Nathan G Lawler (ORCID 0000-0001-9649-425X)^1,3^

1. Australian National Phenome Centre, Health Futures Institute, Harry Perkins Institute of Medical Research, Murdoch University, Perth, Western Australia 6150, Australia.
2. Chemistry Department, Universidad del Valle, Melendez 76001, Cali, Colombia.
3. Centre for Computational and Systems Medicine, Health Futures Institute, Harry Perkins Institute, Murdoch University, Perth, Western Australia 6150, Australia.
4. Department of Metabolism, Digestion and Reproduction, Faculty of Medicine, Imperial College, London SW7 2AZ, United Kingdom.
5. School of Allied Health, College of Health and Education, Murdoch University, Perth, Western Australia 6150, Australia.
6. Centre for Healthy Ageing, Health Futures Institute, Murdoch University, Perth, Western Australia 6150, Australia.

***Corresponding author:** Maartje Cox ([maartje.cox@murdoch.edu.au](mailto:maartje.cox@murdoch.edu.au)); Australian National Phenome Centre, Harry Perkins Institute of Medical Research, 5 Robin Warren Drive, Murdoch WA 6150, Australia

**Content:**

**Supplementary Information S1:** Prompt 1 – Original Research Prompt

**Supplementary Information S2:** Prompt 2 – Human Participants Prompt

**Supplementary Information S3:** Prompt 3 – Inclusion Criteria Prompt

**Supplementary Information S4:** Prompt 4 – Cohort RoB Prompt

**Supplementary Information S5:** Prompt 5 – Reporting RoB Prompt

**Supplementary Information S6:** Prompt 6 – Metabolites RoB Prompt

**Supplementary Information S7:** Prompt 7 – Samples RoB Prompt

**Supplementary Information S8:** Prompt 8 – Analysis RoB Prompt

**Supplementary Information S9:** Prompt 9 – Magnet RoB Prompt

**Supplementary Information S10:** Prompt 10 – Design Prompt

**Supplementary Information S11:** Prompt 11 – Symptoms Prompt

**Supplementary Information S12:** Prompt 12 – Demographics Prompt

**Supplementary Information S13:** Prompt 13 – Infection History Prompt

**Supplementary Information S14:** Prompt 14 – Sample Analysis Prompt

**Supplementary Information S15:** PubMed Search String

**Supplementary Information S16:** Rationale for modifications to published RoB tool

**Fig. S1** Confusion matrices indicating agreement between AI and gold standard in evaluating the risk of bias of included studies

**Supplementary Information S1:** Prompt 1 – Original Research Prompt

Task:

Read the provided title and abstract.

Instructions:

original: Based on the title and abstract, identify whether the article presents new data (i.e., is original research). If the article describes new data collection (e.g., experimental methods, data collection, and results), return "Yes". If the article synthesises existing literature (e.g., reviews, which may be labelled as "literature review", "systematic review", "scoping review", "narrative review", or similar), return "No". If the article uses data from a public database or from previously published research, return "No". If other methods were used but it is clear that no new data were collected in the study, return "No". If it is unclear whether new data were collected, return "Not sure".

reason: Provide a brief explanation for the answer provided under "original" based on information provided in the abstract.

Requirements:

Return a JSON object with this exact structure:

{

"original": "Yes/No/Not sure",

"reason": "concise explanation based on abstract"

}

Replace values with the appropriate answers based on the title and abstract.

Ensure that the output only contains the JSON object, without additional text, comments, or formatting.

**Supplementary Information S2:** Prompt 2 – Human Participants Prompt

Task overview:

Read the provided title and abstract.

Instructions:

Determine whether this study performed research on a group of human participants (e.g., described as "patients", "participants", "females, "males", "children", "adults", "elderly", or in another way where it is clear that the subjects under investigation were human, and not animals, plants, cell cultures, or other non-human subjects).

human: If the study performed research on a group of human participants, return "Yes". If not, return "No". If it is unclear whether the study performed research on humans, return "Not sure".

Requirements:

Return a JSON object with this exact structure:

{

"human": "Yes/No/Not sure",

"reason": "concise explanation based on abstract"

}

Replace values with the appropriate answers based on the title and abstract.

Ensure that the output only contains the JSON object, without additional text, comments, or formatting.

**Supplementary Information S3:** Prompt 3 – Inclusion Criteria Prompt

Task overview:

Read the provided article.

Instructions:

Determine whether the provided article meets the following two criteria based only on its content.

population: If the study population includes both Long-COVID patients (also called post-COVID, post-acute sequelae of SARS-CoV-2, post-acute sequelae of COVID-19, PACS, or PASC patients) and healthy controls, or Long-COVID patients and recovered SARS-CoV-2 individuals, return "Yes". If Long-COVID patients or a healthy/recovered group are not included, or if it includes only acute COVID-19 patients and controls/recovered individuals, return "No". If the study does not explicitly report this information, return "Not reported".

metabolicPhenotype: If the article investigates the metabolic phenotype (also called the metabolic profile, metabolome, metabonome, or phenome) of the study population using techniques such as nuclear magnetic resonance (NMR) spectroscopy or mass spectrometry (including variations like LC-MS, UHPLC-MS, HPLC-MS, MS/MS, LC-MS/MS, UHPLC-MS/MS, or GC-MS), return "Yes". If not, return "No". Important: if the study used mass spectrometry and/or NMR methods only for proteomics, lipidomics, transcriptomics, or glycomics, without metabolomics, return "No". If it is not explicitly reported whether the study used metabolic phenotyping methods, return "Not reported".

Requirements:

Provide an answer for each of the two criteria.

Justify each answer in 1-2 sentences in the `reason` field.

Return a JSON object with this exact structure:

[

{

"criterion": "population",

"answer": "Yes/No/Not reported",

"reason": "concise explanation based on article content"

},

{

"criterion": "metabolicPhenotype",

"answer": "Yes/No/Not reported",

"reason": "concise explanation based on article content"

}

]

Replace values with the appropriate answers based on the article's content.

Ensure that the output only contains the JSON object, without additional text, comments, or formatting.

**Supplementary Information S4:** Prompt 4 – Cohort RoB Prompt

Task overview:

Read the provided article.

Instructions:

Assess whether the article meets the five criteria below based only on the article’s content:

longCovidNumber: If 50 or more Long-COVID patients (also called post-COVID, post-acute sequelae of SARS-CoV-2, post-acute sequelae of COVID-19, PACS, or PASC patients) were included in the study, return "1". If 10 to 49 Long-COVID patients were included, return "0". If < 10 Long-COVID patients were included, return "-1".

symptomDuration: If Long-COVID patients participating in the study experienced symptoms for at least 2 months after testing positive for the SARS-CoV-2 virus, return "1". If not, or if it is not reported for how long Long-COVID patients participating in the study experienced symptoms after testing positive for the SARS-CoV-2/COVID-19 virus, return "-1".

infection: If SARS-CoV-2 infection in Long-COVID patients was confirmed by a positive PCR, SARS-CoV-2 rapid antigen, or antibody test, return "1". If not, or if it is not reported whether SARS-CoV-2 infection in Long-COVID patients was confirmed by a positive PCR, SARS-CoV-2 rapid antigen, or antibody test, return "-1".

definitionRecovered: If the study did not include a group of individuals who recovered from infection with the SARS-CoV-2 virus, return "NA" for this criterion and continue to answer the remaining criteria. If the study included a group of individuals who recovered from infection with the SARS-CoV-2 virus and if their SARS-CoV-2 infection was confirmed by a positive PCR, SARS-CoV-2 rapid antigen, or antibody test, return "1". If not, or if it is not reported whether the recovered individuals' SARS-CoV-2 infection was confirmed by a positive PCR, SARS-CoV-2 rapid antigen, or antibody test, return "-1".

recovered: If the study did not include a group of individuals who recovered from infection with the SARS-CoV-2 virus, return "NA" for this criterion. If the study included a group of individuals who recovered from infection with the SARS-CoV-2 virus and if this group is defined as those who tested positive for SARS-CoV-2 but did not experience any SARS-CoV-2-induced symptoms anymore at the time of study participation, return "1". If not, or if it is not reported how the group of recovered individuals is defined, return "0".

Requirements:

Provide an answer for each of the five criteria.

Justify each score in 1-2 sentences in the `value` field.

Return a JSON object with this exact structure:

[

{

"parameter": "longCovidNumber",

"score": "1/0/-1",

"value": "number of Long-COVID patients included in the study"

},

{

"parameter": "symptomDuration",

"score": "1/-1",

"value": "duration of symptoms in Long-COVID patients"

},

{

"parameter": "infection",

"score": "1/-1",

"value": "evidence for SARS-CoV-2 confirmation in Long-COVID group"

},

{

"parameter": "definitionRecovered",

"score": "1/-1/NA",

"value": "evidence for SARS-CoV-2 confirmation in recovered group"

},

{

"parameter": "recovered",

"score": "1/0/NA",

"value": "definition of recovered group"

}

]

Replace values with the appropriate answers based on the article's content.

Ensure that the output only contains the JSON object, without additional text, comments, or formatting.

**Supplementary Information S5:** Prompt 5 – Reporting RoB Prompt

Task overview:

Read the provided article.

Instructions:

Assess whether the article meets the five criteria below based only on the article’s content:

validationCohort: If the study validated their metabolomics results using an independent internal or external validation cohort, return "1". If not or not reported, return "0".

characteristicsReported: If participant characteristics (e.g., age, BMI, sex) were reported separately per study/participant group (e.g., Long-COVID and healthy controls), return "1". If not, return "-1".

characteristicsPerCohort: If the study validated their metabolomics results using an independent internal or external cohort and participant characteristics (e.g., age, BMI, sex) are reported for each cohort (i.e., discovery and validation cohort) separately, return "1". If a validation cohort was used but characteristics were not reported per cohort, return "-1". If no validation cohort was included, return "NA".

inclusionCriteria: If the in- and/or exclusion criteria are stated per study/participant group, return "1". If not, return "0".

confoundersReported: If potential confounders (e.g., sex, age, BMI) are compared between groups and, if significant, accounted for in the data analysis (e.g., by adding confounders to models or by stratifying analyses), return "1". If potential confounders are compared between groups but not considered in data analysis by adding them to models or by stratifying analyses even if they differed significantly between groups, return "-1". If potential confounders are not compared between groups and not considered for data analysis, return "-1".

Requirements:

Provide an answer for each of the five criteria.

Justify each score in 1-2 sentences in the `value` field.

Return a JSON object with this exact structure:

[

{

"parameter": "validationCohort",

"score": "1/0",

"value": "brief description of validation cohort (internal/external or not used)"

},

{

"parameter": "characteristicsReported",

"score": "1/-1",

"value": "list of all participant characteristics reported per study group"

},

{

"parameter": "characteristicsPerCohort",

"score": "1/-1/NA",

"value": "list of all participant characteristics reported per cohort (i.e., discovery cohort and validation cohort)"

},

{

"parameter": "inclusionCriteria",

"score": "1/0",

"value":

[

{

"name": "group 1 name",

"inclusionCriteria": "list of all inclusion criteria applied to group 1",

"exclusionCriteria": "list of all exclusion criteria applied to group 1"

},

{

"name": "group 2 name",

"inclusionCriteria": "list of all inclusion criteria applied to group 2",

"exclusionCriteria": "list of all exclusion criteria applied to group 2"

}

]

},

{

"parameter": "confoundersReported",

"score": "1/-1",

"value": "list of all confounders discussed and/or included in analyses"

}

]

Replace values with the appropriate answers based on the article's content.

Ensure that the output only contains the JSON object, without additional text, comments, or formatting.

**Supplementary Information S6:** Prompt 6 – Metabolites RoB Prompt

Task overview:

Read the provided article.

Instructions:

Use the predefined background information provided: refmsi('2.9') refmsi('2.9.1'): refmsi('2.9.2') refmsi('2.9.3') refmsi('2.9.4')

Assess whether the level of metabolite identification for the metabolites reported in the provided article should be classified as level 1, level 2, level 3, or level 4 using these criteria:

Metabolites should be classified as level 1 if they meet the following criteria: refmsi('2.9.1'): refmsi('2.9.6') refmsi('2.9.7') refmsi('2.9.8').

Metabolites should also be classified as level 1 if they used the Bruker IVDR method for their NMR analysis.

Metabolites should be classified as level 2 if they do not meet level 1 criteria, but do meet the following criterion: refmsi('2.9.2')

Metabolites should be classified as level 3 if they do not meet level 1 or 2 criteria, but do meet the following criterion: refmsi('2.9.3')

Metabolites should be classified as level 4 if they do not meet level 1, 2, or 3 criteria, but do meet the following criterion: refmsi('2.9.4')

Metabolites should be classified as NA if the article does not report the method of metabolite identification in sufficient detail to determine which level of metabolite identification is appropriate.

Requirements:

Provide the metabolite identification level.

Justify the answer in 1-2 sentences.

Return a JSON object with this exact structure:

{

"metaboliteIdentificationLevel": "level 1/level 2/level 3/level 4/NA",

"explanation": "Reason for classification level based on the criteria provided"

}

Replace values with the appropriate answers ("level 1", "level 2", "level 3", "level 4", or "NA") based on the article's content.

Ensure that the output only contains the JSON object, without additional text, comments, or formatting.

**Supplementary Information S7:** Prompt 7 – Samples RoB Prompt

Task overview:

Read the provided article.

Instructions:

Assess whether the article meets the five criteria below, focusing only on metabolomics analysis of samples from Long-COVID patients (also called post-COVID, post-acute sequelae of SARS-CoV-2, post-acute sequelae of COVID-19, PACS, or PASC patients), based on the article’s content:

randomisation: If samples were randomized prior to analyses, return "1". If not or if not reported, return "0".

qualityControl: If a pooled quality control (QC) sample was used and if its reproducibility was shown, return "1". If a pooled quality control sample was used but its reproducibility was not shown, return "0". If no pooled quality control sample was used or if it is not reported whether a pooled quality control sample was used, return "-1". If the study did not analyse biofluid samples from Long-COVID patients (e.g., in 1H MRS research), return "NA".

dataAnalysis: If it is that clearly described how metabolomics data were processed and which (statistical) methods were used to analyse the data, that the protocol is reproducible, return "1". If some information was provided regarding the data analysis workflow, but there are some unclarities and the level of detail is insufficient to reproduce the data analysis workflow, return "0". If no information regarding the data analysis workflow was provided at all, return "-1".

software: If an established software was used for metabolomics data analysis or if a newly developed algorithm was provided and validated for data analysis, return "1". If new software or algorithm was used but the information provided on the software or algorithm was incomplete and/or not validated, return "0". If a new software or algorithm was used but no information on the software or algorithm was provided at all and/or the new software or algorithm was not validated, return "-1".

significanceLevel: If the levels of significance were corrected for multiple testing (e.g., with Bonferroni or Benjamini-Hochberg correction), return "1". If the levels of significance were not corrected for multiple testing or if it is not reported whether significance levels were corrected for multiple testing, return "-1".

Requirements:

Provide an answer for each of the five criteria.

Justify each score in 1-2 sentences in the `value` field.

Return a JSON object with this exact structure:

[

{

"parameter": "randomisation",

"score": "1/0",

"value": "brief justification of answer"

},

{

"parameter": "qualityControl",

"score": "1/0/-1/NA",

"value": "brief justification of answer"

},

{

"parameter": "dataAnalysis",

"score": "1/0/-1",

"value": "brief justification of answer"

},

{

"parameter": "software",

"score": "1/0/-1",

"value": "list with names of all software tools used for data analysis"

},

{

"parameter": "significanceLevel",

"score": "1/-1",

"value": "list of all methods used to correct for multiple testing (if any)"

}

]

Replace values with the appropriate answers based on the article's content.

Ensure that the output only contains the JSON object, without additional text, comments, or formatting.

**Supplementary Information S8:** Prompt 8 – Analysis RoB Prompt

Task overview:

Read the provided article.

Instructions:

Step 1:

Determine whether the study analysed biological samples collected from Long-COVID patients (also called post-COVID, post-acute sequelae of SARS-CoV-2, post-acute sequelae of COVID-19, PACS, or PASC patients) **specifically for metabolomics analyses**.

- If not, return a JSON object with all parameters set to "score": "NA" and value: "no biological samples from Long-COVID patients were used for metabolomics analysis".

- If yes, proceed to Step 2.

Step 2:

Evaluate whether the article meets the five criteria below. Only consider biological samples from Long-COVID patients (also called post-COVID, post-acute sequelae of SARS-CoV-2, post-acute sequelae of COVID-19, PACS, or PASC patients) used for metabolomics purposes.

sampleType: Determine whether the article reports which biological sample types (e.g., plasma, serum, saliva, cerebrospinal fluid, breath condensate, faeces, urine) were collected from Long-COVID patients for metabolomics. If the study reports which biological sample types were collected from Long-COVID patients for metabolomics analysis, return "1". If not explicitly reported, return "-1".

sampleCollection: Determine whether the article reports any of the following parameters regarding the collection of biological samples used for metabolomics analysis from Long-COVID patients: 1. collection method (e.g., venipuncture, or needle biopsy for tissue collection), 2. collection tubes (e.g., standard tubes, EDTA tubes, heparin tubes), 3. use of anticoagulants. If the study reports how biological samples were taken from Long-COVID patients for metabolomics analysis by describing one or more of these 3 collection-related details, return "1". If none of these 3 collection-related details are explicitly reported, return "-1".

samplePreparation: Determine whether the article reports any of the following parameters regarding laboratory sample processing for metabolomics analysis: 1. centrifugation, 2. use of solvents, 3. use of buffers, 4. resolubilization, 5. derivatization, 6. resuspension, 7. addition of internal standards, 8. addition of calibrants, 9. tissue handling (e.g., lyophilization, pulverization/homogenization). If the study describes how Long-COVID samples were processed in the laboratory before metabolomics analysis by describing one or more of these 9 preparation-related details, return "1". If none of these 9 preparation-related details are explicitly reported, return "-1".

separation: If the study did not use chromatography to analyse Long-COVID samples for metabolomics purposes, return "NA" and proceed with answering the next question. If the study used chromatography methods and reports which type of chromatography (e.g., liquid chromatography (LC) or gas chromatography (GC)) was used, return "1". If the chromatography type is not explicitly reported, return "-1".

massSpec: If the study did not use mass spectrometry to analyse Long-COVID samples for metabolomics purposes, return "NA" and proceed with answering the next question. Examples of mass spectrometry types include quadrupole, triple quadrupole, time of flight (TOF), ion trap, and orbitrap. If the study used mass spectrometry methods and reports which mass spectrometry instrument was used by reporting either the type of mass spectrometry or the instrument's commercial name, or both, return "1". If the MS instrument type or commercial name is not explicitly reported, return "-1".

Requirements:

Provide an answer for each of the five criteria.

Justify each score in 1-2 sentences in the `value` field.

Return a JSON object with this exact structure:

[

{

"parameter": "sampleType",

"score": "1/-1/NA",

"value": "sample type 1; sample type 2"

},

{

"parameter": "sampleCollection",

"score": "1/-1/NA",

"value": "brief justification of answer"

},

{

"parameter": "samplePreparation",

"score": "1/-1/NA",

"value": "brief justification of answer"

},

{

"parameter": "separation",

"score": "1/-1/NA",

"value": "brief justification of answer"

},

{

"parameter": "massSpec",

"score": "1/-1/NA",

"value": "brief justification of answer"

}

]

Replace values with the appropriate answers based on the article's content.

Ensure that the output only contains the JSON object, without additional text, comments, or formatting.

**Supplementary Information S9:** Prompt 9 – Magnet RoB Prompt

Task overview:

Read the provided article.

Instructions:

Determine whether the study used nuclear magnetic resonance (NMR) spectroscopy, magnetic resonance spectroscopy (MRS), or magnetic resonance imaging (MRI) to analyse samples from Long-COVID patients (also called post-COVID, post-acute sequelae of SARS-CoV-2, post-acute sequelae of COVID-19, PACS, or PASC patients) or to analyse Long-COVID patients in vivo for metabolomics purposes.

If the study did not use NMR, MRI, or MRS, return {"parameter": "nmr","score": "NA","value": "The study did not use NMR, MRS, or MRI for the analysis of Long-COVID samples or in vivo analysis of Long-COVID patients for metabolomics"}

magnet: If the study used nuclear magnetic resonance (NMR) spectroscopy, magnetic resonance spectroscopy (MRS), or magnetic resonance imaging (MRI) and reports either the instrument's magnetic field strength or the instrument's proton resonance frequency, or both, return "1". If not, return "-1".

Requirements:

Provide an answer for the criterion.

Justify the score in 1-2 sentences in the `value` field.

Return a JSON object with this exact structure:

{

"parameter": "magnet",

"score": "1/-1/NA",

"value": "brief justification of answer"

}

Replace values with the appropriate answers based on the article's content.

Ensure that the output only contains the JSON object, without additional text, comments, or formatting.

**Supplementary Information S10:** Prompt 10 – Design Prompt

Task overview:

Read the provided article.

Instructions:

Use the article provided and extract relevant details from the article to provide the following three parameters:

design: Identify and extract the type of study design used in the article (e.g., cross-sectional, case-control, randomized-controlled trial, longitudinal).

country: Extract the country where the study was conducted.

participants: Extract the name of each participant group and the number of participants in each group.

Requirements:

Provide an answer for each of the three parameters.

If the article does not explicitly report a given detail, return "NA".

Return a JSON object with this exact structure:

{

"design": "study design",

"country": "country in which the study was conducted",

"participants": [

{

"name": "name of group",

"n": "number of participants in group"

}

]

}

Replace values with the appropriate answers based on the article's content.

Additional instructions:

If the article does not explicitly report a given detail, return "NA".

Maintain exact terminology from the article.

Ensure that the JSON output is properly formatted, without any additional text, comments, or explanations.

**Supplementary Information S11:** Prompt 11 – Symptoms Prompt

Task overview:

Read the provided article.

Instructions:

Use the article provided and extract relevant details from the article to provide the following information:

symptoms: Extract the symptoms that were reported by Long-COVID participants (also called post-COVID, post-acute sequelae of SARS-CoV-2, post-acute sequelae of COVID-19, PACS, or PASC patients) taking part in this study. For each symptom, include:

Name of the symptom

Number of Long-COVID participants who reported this symptom

Percentage of Long-COVID participants who reported this symptom

Symptom severity (include central tendency and variability if provided; if only a qualitative description is available, report it as is)

P-value of the comparison between symptom severity of Long-COVID patients and healthy controls

Requirements:

Provide an answer for all parameters per symptom reported.

If the article does not explicitly report a given detail, return "NA".

Important: only extract those symptoms that were reported by Long-COVID participants who took part in this study.

Return a JSON object with this exact structure:

{

"symptoms": [

{

"name": "name symptom 1",

"nReportedBy": "number of Long-COVID patients who reported this symptom",

"percentageReportedBy": "percentage of Long-COVID patients who reported this symptom",

"metric": "type of central tendency reported (i.e., mean or median)",

"value": "mean or median severity of this symptom OR qualitative description of the severity of the symptom",

"deviation": "standard deviation, interquartile range [quartile 1, quartile 3], or range (min, max) of symptom severity",

"pValue": "p-value of the symptom when comparing Long-COVID to healthy controls"

}

]

}

Replace values with the appropriate answers based on the article's content.

Additional instructions:

If the article does not explicitly report a given detail, return "NA".

Maintain exact terminology from the article.

If a symptom's severity is only qualitatively described, return the exact qualitative description.

Ensure that the JSON output is properly formatted, without any additional text, comments, or explanations.

**Supplementary Information S12:** Prompt 12 – Demographics Prompt

Task overview:

Read the provided article.

Instructions:

Use the article provided and extract relevant details from the article to provide the following information:

demographics: Extract for each participant group:

age: Extract the ages of participants in each group (central tendency, variability, unit).

sex: Extract the number and/or percentage of male and female participants in each group.

height: Extract the heights of participants in each group (central tendency, variability, unit).

weight: Extract the weights of participants in each group (central tendency, variability, unit).

bmi: Extract the BMIs of participants in each group (central tendency, variability, unit).

Requirements:

Provide an answer for each of the five parameters per participant group.

If the article does not explicitly report a given detail, return "NA".

Return a JSON object with this exact structure:

{

"demographics": [

{

"name": "name of group 1",

"age": {

"metric": "type of central tendency reported (i.e., mean or median)",

"value": "mean or median age of group 1",

"deviation": "standard deviation, interquartile range [quartile 1, quartile 3], or range (min, max) of age of group 1",

"unit": "unit in which age is presented in the article"

},

"sex": {

"number": "number and/or percentage of male and female participants in group 1"

},

"height": {

"metric": "type of central tendency reported (i.e., mean or median)",

"value": "mean or median height of group 1",

"deviation": "standard deviation, interquartile range [quartile 1, quartile 3], or range (min, max) of height of group 1",

"unit": "unit in which height is presented in the article"

},

"weight": {

"metric": "type of central tendency reported (i.e., mean or median)",

"value": "mean or median weight of group 1",

"deviation": "standard deviation, interquartile range [quartile 1, quartile 3], or range (min, max) of weight of group 1",

"unit": "unit in which weight is presented in the article"

},

"bmi": {

"metric": "type of central tendency reported (i.e., mean or median)",

"value": "mean or median BMI of group 1",

"deviation": "standard deviation, interquartile range [quartile 1, quartile 3], or range (min, max) of BMI of group 1",

"unit": "unit in which BMI is presented in the article"

}

}

]

}

Replace values with the appropriate answers based on the article's content.

Additional instructions:

If the article does not explicitly report a given detail, return "NA".

Maintain exact terminology from the article.

Ensure that the JSON output is properly formatted, without any additional text, comments, or explanations.

**Supplementary Information S13:** Prompt 13 – Infection History Prompt

Task overview:

Read the provided article.

Instructions:

Use the article provided and extract relevant details from the article to provide the following information:

infectionHistory: For each participant group, extract:

Time between the latest infection with the SARS-CoV-2 (COVID-19) virus and participation in the study (central tendency, variability, unit)

Number of infections with the SARS-CoV-2 (COVID-19) virus (central tendency, variability)

Requirements:

Provide an answer for each of the two parameters per participant group.

If the article does not explicitly report a given detail, return "NA".

If a parameter does not apply to a certain participant group (e.g., healthy control participants who had never been infected with SARS-CoV-2), return "NA".

Return a JSON object with this exact structure:

{

"infectionHistory": [

{

"name": "name of group 1",

"timeSinceInfection": {

"metric": "type of central tendency reported (i.e., mean or median)",

"value": "mean or median time since latest infection with the SARS-CoV-2 (COVID-19) virus",

"deviation": "standard deviation, interquartile range [quartile 1, quartile 3], or range (min, max) of the time since infection with the SARS-CoV-2 (COVID-19) virus for group 1",

"unit": "unit in which time since infection with the SARS-Cov-2 (COVID-19) virus is presented in the article"

},

"numberOfInfections": {

"metric": "type of central tendency reported (i.e., mean or median)",

"value": "mean or median number of infections with the SARS-CoV-2 (COVID-19) virus",

"deviation": "standard deviation, interquartile range [quartile 1, quartile 3], or range (min, max) of the number of infections with the SARS-CoV-2 (COVID-19) virus",

}

}

]

}

Replace values with the appropriate answers based on the article's content.

Additional instructions:

If the article does not explicitly report a given detail, return "NA".

Maintain exact terminology from the article.

If infection history is only qualitatively described, return the exact qualitative description.

Ensure that the JSON output is properly formatted, without any additional text, comments, or explanations.

**Supplementary Information S14:** Prompt 14 – Sample Analysis Prompt

Task overview:

Manually screen the article and extract relevant details to evaluate each of the following criteria separately.

If a criterion is not explicitly reported in the article, return "NA".

sample: Extract the biological sample types collected from Long-COVID patients (also called post-COVID, post-acute sequelae of SARS-CoV-2, post-acute sequelae of COVID-19, PACS, or PASC patients) that were used for metabolomics analysis (e.g., urine, blood plasma, blood serum, muscle tissue, saliva, cerebrospinal fluid).

metabolomics: Determine whether the study used a hypothesis-driven (targeted) or hypothesis-generating (untargeted) metabolomics approach. Report "Hypothesis-driven" if the study was hypothesis-driven, focusing on the measurement or quantification of specific metabolites. Record "Hypothesis-generating" if it was an exploratory analysis without predefined target metabolites.

analyticalPlatform: Extract the exact metabolomics analytical platforms used for data acquisition in the study (e.g., NMR, LC-MS, UHPLC-MS, HPLC-MS, MS/MS, LC-MS/MS, UHPLC-MS/MS, or GC-MS) as well as the instrument name and manufacturer name (e.g., NMR (Bruker IVDr Avance III HD 600 MHz system) or UHPLC-MS (Waters Acquity UHPLC, Bruker Impact IITM Ultra-High Resolution Qq-Time-Of-Flight MS)).

Output format:

Return a nested JSON object with this exact structure:

{

"sample": ["sample type 1", "sample type 2", "sample type 3"],

"metabolomics": "Hypothesis-driven/targeted metabolomics or Hypothesis-generating/untargeted metabolomics",

"analyticalPlatform": [

"metabolomics analytical platform 1 (Instrument name, Manufacturer)",

"metabolomics analytical platform 2 (Instrument name, Manufacturer)",

"metabolomics analytical platform 3 (Instrument name, Manufacturer)"

]

}

Replace values with the appropriate answers based on the article's content.

Additional instructions:

If the article does not explicitly report a given detail, return "NA".

Maintain exact terminology from the article.

Ensure that the JSON output is properly formatted, without any additional text, comments, or explanations.

**Supplementary Information S15:** PubMed Search String

(“Post-Acute COVID-19 Syndrome”[MeSH Terms] OR “long COVID”[All Fields] OR “long-COVID”[All Fields] OR “long COVID-19”[All Fields] OR “long-COVID-19”[All Fields] OR “long-COVID syndrome”[All Fields] OR “long COVID syndrome”[All Fields] OR “post-COVID”[All Fields] OR “post-COVID syndrome”[All Fields] OR “post-acute COVID-19 syndrome”[All Fields] OR “PACS”[All Fields] OR “post-COVID-19 syndrome”[All Fields] OR “post COVID-19 syndrome”[All Fields] OR “post-acute sequelae of SARS-CoV-2”[All Fields] OR “post-acute sequelae of COVID-19”[All Fields] OR “postacute sequelae of COVID-19”[All Fields] OR “postacute coronavirus”[All Fields] OR “PASC”[All Fields])

AND

(“Metabolomics”[MeSH Terms] OR “Metabolome”[MeSH Terms] OR “metabolic phenotyping”[All Fields] OR “metabolomic*”[All Fields] OR “metabolome-wide”[All Fields] OR “metabolic profiling”[All Fields] OR “metabonomic*”[All Fields] OR “metabonome”[All Fields] OR “metabolite*”[All Fields] OR “Mass Spectrometry”[MeSH Terms] OR “mass spectrometry”[All Fields] OR “LCMS”[All Fields] OR “LC-MS”[All Fields] OR “GCMS”[All Fields] OR “GC-MS”[All Fields] OR “UPLCMS”[All Fields] OR “UPLC-MS”[All Fields] OR “HPLC-MS”[All Fields] OR “LC-MS/MS”[All Fields] OR “UPLC-MS/MS”[All Fields] OR “Magnetic Resonance Spectroscopy”[MeSH Terms] OR “nuclear magnetic resonance spectroscopy”[All Fields] OR “NMR spectroscopy”[All Fields] OR “nuclear magnetic resonance”[All Fields] OR “NMR”[All Fields])

**Supplementary Information S16:** Modifications and rationales for modifications of published RoB tool

| **Item in the tool by U et al. (2022)** | **Item in the newly proposed tool** | **Modification** | **Rationale** |
| --- | --- | --- | --- |
| **Domain 1 – Study Design** | | | |
| 1.1 | 1.1 | Changed hepatocellular carcinoma to Long Covid | Population of interest for current review is Long Covid rather than HCC |
| 1.2 | 1.2a and 1.2b | Separated criterion 1.2 into two criteria | The original criterion 1.2 evaluated two components. For clarity, these two components are now separated over two criteria, allowing for the evaluation of each |
| 1.3 | 1.3, 1.4, 1.5, 1.6 | Tailored diagnostic criteria to Long Covid | The population of interest for the current review consists of Long Covid patients, people recovered from infection with SARS-CoV-2 without persisting symptoms, and healthy controls. Criteria relevant for the correct identification of the population of interest were added, while ensuring that only one component was evaluated under each criterion |
| 1.4 | 1.7 | Added “…per study group” | Different in- and exclusion criteria apply to patients and controls, the modification specified that the reporting of in- and exclusion criteria are evaluated separately per study population |
| 1.5 | 1.8 | No modification |  |
| 1.6 | 1.2 | No modification |  |
| **Domain 2 – Chemical analysis** | | | |
|  | 2.1 | New criterion | The reporting of the sample type is relevant as there are differences in the types of metabolites that can be detected – and their concentrations – between different biofluids |
| 2.1 | 2.2 | Specified which aspects of sample collection should be described in order to yield Low RoB | The tool by U et al. did not specify how “no concern” was defined, and thus, when a Low or Medium RoB score was appropriate. The modification allows for a more objective determination of the appropriate RoB score |
| 2.2 | 2.4 | No modification |  |
| 2.3 | 2.3 | Specified which aspects of sample processing should be stated in order to yield Low RoB | The tool by U et al. did not specify how “no concern”, “minor concern”, and “major concern” were defined. The modification allows for an objective determination of the correct RoB score |
| 2.4 | 2.5 | No modification |  |
| 2.5 | 2.6, 2.7, 2.8 | Separated criterion 2.5 into three criteria and objectified response categories | The original criterion referred to studies by Sumner et al., 2007 and by Goodacre et al., 2007, without specifying which criteria of these studies were considered in determining the RoB score or how “no concern”, “minor concern”, and “major concern” were defined. The modification evaluates the reporting of chromatography, mass spectrometry, and magnetic resonance spectroscopy methods separately, and specifies when a Low or High RoB score is appropriate |
| **Domain 3 – Data Analysis** | | | |
| 3.1 | 3.1 | Specified when criterion should be rated as Low, Medium, or High RoB | The original criterion did not detail when the item should be scored as Low, Medium, or High RoB |
| 3.2 | 3.2 | No modification |  |
| 3.3 | 3.3 | No modification |  |
| **Domain 4 – Metabolite Identification** | | | |
| 4.1 | 4.1 | No modification |  |
| 4.2 |  | Removed criterion | As we performed the RoB assessment on a methodological level rather than on a metabolite level, this criterion became unapplicable |
| 4.3 |  | Removed criterion | As we performed the RoB assessment on a methodological level rather than on a metabolite level, this criterion became unapplicable |

**Fig. S1** Confusion matrices indicating agreement between AI and gold standards in evaluating the risk of bias of included studies. Rows indicate the composition of the manually created gold standard categories, while columns indicate how predicted categories were composed. Values along the diagonal (highlighted in dark blue) indicate the number of articles for which there was agreement between the gold standards and the responses generated by AI
